# Neuronal Target Identification Requires AHA-1-Mediated Fine-Tuning of Wnt Signaling in *C. elegans*

**DOI:** 10.1371/journal.pgen.1003618

**Published:** 2013-06-27

**Authors:** Jingyan Zhang, Xia Li, Angela R. Jevince, Liying Guan, Jiaming Wang, David H. Hall, Xun Huang, Mei Ding

**Affiliations:** 1 State Key Laboratory of Molecular Developmental Biology, Institute of Genetics and Developmental Biology, Chinese Academy of Sciences, Beijing, China; 2 University of Chinese Academy of Sciences, Beijing, China; 3 Department of Neuroscience, Albert Einstein College of Medicine, New York, New York, United States of America; Stanford University School of Medicine, United States of America

## Abstract

Electrical synaptic transmission through gap junctions is a vital mode of intercellular communication in the nervous system. The mechanism by which reciprocal target cells find each other during the formation of gap junctions, however, is poorly understood. Here we show that gap junctions are formed between BDU interneurons and PLM mechanoreceptors in *C. elegans* and the connectivity of BDU with PLM is influenced by Wnt signaling. We further identified two PAS-bHLH family transcription factors, AHA-1 and AHR-1, which function cell-autonomously within BDU and PLM to facilitate the target identification process. *aha-1* and *ahr-1* act genetically upstream of *cam-1*. CAM-1, a membrane-bound receptor tyrosine kinase, is present on both BDU and PLM cells and likely serves as a Wnt antagonist. By binding to a cis-regulatory element in the *cam-1* promoter, AHA-1 enhances *cam-1* transcription. Our study reveals a Wnt-dependent fine-tuning mechanism that is crucial for mutual target cell identification during the formation of gap junction connections.

## Author Summary

The establishment of functional neuronal circuits requires that different neurons respond selectively to guidance molecules at particular times and in specific locations. In the target region, where cells connect, the same guidance molecules steer the growth of neurites from both the neuron and its target cell. The spatial, temporal, and cell-type-specific regulation of neuronal connection needs to be tightly regulated and precisely coordinated within the neuron and its target cell to achieve effective connection. In this study, we found that the precise connectivity of the BDU interneuron and the PLM mechanoreceptor in the nematode worm *Caenorhabditis elegans* is influenced by Wnt signaling. BDU-PLM contact also depends on the transcription factor AHA-1, which functions within both BDU and PLM cells to enhance transcription of the gene encoding the trans-membrane receptor CAM-1. CAM-1 is present on BDU and PLM and likely serves as a Wnt antagonist, thus linking transcriptional regulation by AHA-1 to modulation of Wnt signaling. Therefore, our study reveals a locally confined, cell type-specific and cell-autonomous mechanism that mediates mutual target identification.

## Introduction

According to the neuron doctrine, the neural network is a linkage of discrete nerve cells, which are connected to one another indirectly through chemical synapses or directly through electrical synapses, also known as gap junctions. To wire the neuronal network, many neurons extend neurites (axons and dendrites) over great distances, bypassing numerous potential but inappropriate targets to reach the correct area. Over the past two decades, a number of important long-range and short-range environmental cues that regulate neurite guidance have been discovered, including Netrins, Semaphorins, Slits and Ephrins [1]. The list of guidance cues was further expanded by the finding that morphogens such as Wnt and Shh can also guide neurites in specific directions [2], [3]

After neurites from both synaptic partners reach the targeting field, a more refined target identification process must occur to establish the final connection. An array of cell adhesion molecules, including cadherins and immunoglobulin super-family proteins, are responsible for the direct selective cell-cell attraction between specific synaptic partner cells [4], [5]. Transcription factors also control synaptic connectivity. For example, the *C. elegans* homeodomain protein UNC-4, together with transcription factors HB9 and Groucho, is involved in the formation of neuronal gap junctions between the interneuron AVA and the motor neuron VA [6]–[9]. A recent study further revealed that UNC-4 antagonizes Wnt signaling to regulate this synaptic choice [10]. In addition, molecules involved in axon guidance play a significant role in the refinement of target identification. During visual system development, for instance, axons of retinal ganglion cells initially project into the optic tectum at incorrect positions along the medial-lateral tectal axis, but later correct this error by altering their trajectory or extending collateral branches at right angles [11]. Ephrins, a class of membrane-bound short-range signaling proteins, are involved in this fine-tuning process [12]. Netrin is a secreted chemotropic factor that can act as an attractive or repellent long-range cue during axon guidance. A recent study showed that the connectivity between AIY and RIA interneurons in *C. elegans* requires the coordination of Netrin-mediated short-range signals in both neurons [13]. In the target region, the same cues may steer neurites from both the pre-synaptic neuron and its target cell. In the formation of neuronal gap junctions, neurites of both reciprocal target cells are in close proximity and are thus very likely under the control of the same environmental signals. Therefore, to achieve effective connectivity, the spatial, temporal, and cell-type-specific responsiveness within both neurites must be tightly regulated and precisely coordinated. However, the detailed mechanism underlying this coordination is largely unknown.

Wnt signaling has recently been linked to axon guidance and synapse patterning [14], [15]. By binding to the extracellular cysteine-rich domain (CRD) of the Frizzled receptor (Frz) and the co-receptor LRP, Wnt activates Dishevelled (Dsh) and triggers downstream events. Receptor tyrosine kinase-like orphan receptor (Ror) proteins also bind Wnt and participate in multiple Wnt-mediated biological processes [16], [17]. In *C. elegans*, CAM-1, the homolog of mammalian Ror2, can function as a Wnt or Frizzled antagonist in cell migration and vulval development, or as a Wnt co-receptor regulating nerve ring organization and axon outgrowth [18]–[21]. In mammals the non-canonical Wnt5a-Ror2-Dsh pathway inhibits the canonical Wnt/β-catenin pathway [22]. The Wnt pathway is also modulated by various activators, including R-spondin and Norrin, and inhibitors, such as DKK, sFRP, WIF, SOST, and Tiki1 [23]. Tight transcriptional regulation of these modulators is important for proper Wnt signaling. For example, the GATA-type transcription factor Trps1 activates expression of the Wnt inhibitors WIF and DKK4 and is essential for vibrissa follicle morphogenesis in mice [24]. Another GATA transcription factor, GATA6, negatively regulates the level of DKK1 in pancreatic cancer [25]. The PAS-bHLH family protein HIF-1 is required for hypoxia-induced transcription of *cam-1* in *C. elegans* [26]. However, the detailed molecular mechanisms underlying transcription-mediated regulation of Wnt activity during neural connection remain to be further elucidated.

Here, we show that *C. elegans* BDU and PLM neurons connect to each other through gap junctions and that Wnt signaling is important for BDU-PLM connectivity. We found that the PAS-bHLH transcription factor AHA-1 and its partner AHR-1 function cell-autonomously within BDU and PLM to regulate expression of the Wnt antagonist CAM-1. Together, our results reveal that transcription-mediated fine-tuning of Wnt signaling is responsible for target identification by BDU and PLM neurons.

## Results

### Development of the BDU-PLM neuronal connection

**Figure 1:**
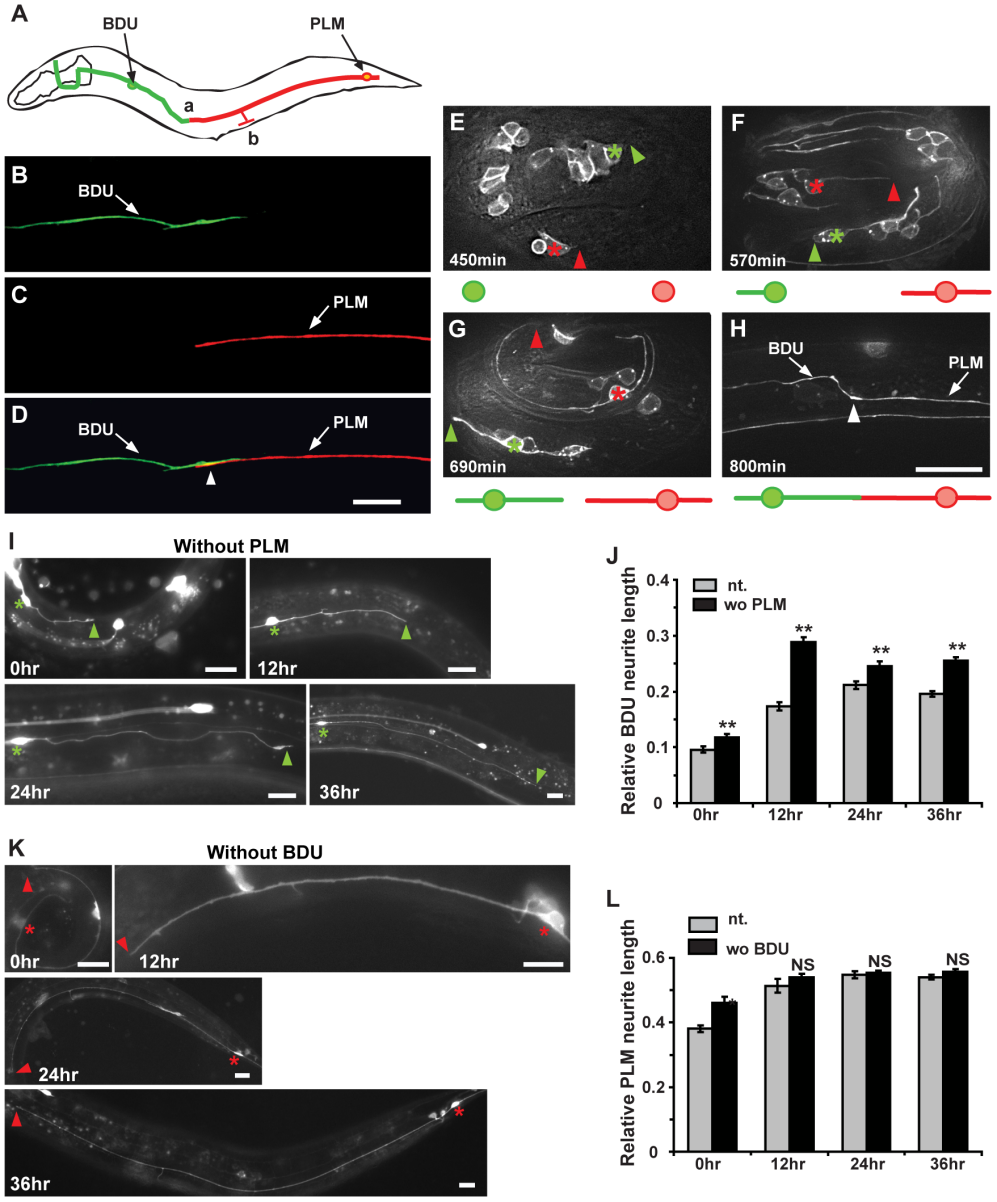
The BDU posterior process connects to the anterior branch of PLM. (A) Schematic drawing of the BDU-PLM connection. The posterior tip of BDU is connected to the anterior branch of PLM at region (a). The PLM branch, where the chemical synapses are localized, is labeled by (b). Arrows point to the BDU and PLM cell bodies. (B–D) P*unc-53*::GFP (green) labels BDU and P*mec-7*::mCherry (red) labels PLM. The arrowhead in (D) indicates where the BDU and PLM neurites meet. (E–H) shows the embryonic development of BDU-PLM connections. (E) BDU (green asterisk) and PLM (red asterisk) neurons are born at the 1.5–2 fold embryonic stage (450 min after the first cell division). The anterior process of PLM extends a significant length, while no posterior process of BDU is noticeable at 570 min (F). At 690 min, both the BDU posterior process and the PLM anterior process are visible (G). By the end of embryonic elongation, the BDU-PLM connection is formed (H). The tips of the BDU posterior process and the PLM anterior process are indicated by green and red arrowheads respectively. The white arrowhead (H) indicates the junction of the BDU posterior process and the PLM anterior process. Below the images are schematic representations of BDU (in green) and PLM (in red). (I) The BDU cell at different developmental stages in the absence of PLM. Green asterisks indicate the BDU cell bodies. Arrowheads point to the tip of the BDU posterior process. (J) Quantification of relative BDU neurite length at different developmental stages in wild type (gray column) or in the absence of PLM cell (black column). The neurite length was measured 0 hr, 12 hr, 24 hr, and 36 hr after hatching. (K) The PLM cell at different developmental stages in the absence of BDU cell. Red asterisks indicate the PLM cell bodies. Arrowheads point to the tip of the PLM posterior process. (L) Quantification of relative PLM neurite length at different developmental stages in wild type (gray column) or in the absence of BDU cell (black column). The neurite length was measured 0 hr, 12 hr, 24 hr, and 36 hr after hatching. Error bars in (J) and (L) represent the standard error of the mean (SEM). ** p<0.01; NS, not significant. Scale bars represent 10 µm.

The BDU neurons are a pair of interneurons with cell bodies situated laterally in the anterior body of *C. elegans*. From its cell body, each BDU neuron projects an anterior process and a posterior process (Figure 1A). Electron microscopy (EM) reconstruction studies have revealed that BDU mainly receives chemical synaptic input from the mechanosensory neurons ALM and AVM through its anterior process around the nerve ring region [27]. However, these EM studies appear to fail to track the BDU posterior process after it turns away from the lateral nerve [27]. Here, using various GFP reporters (Altun and Hall, 2013 in WormAtlas http://www.wormatlas.org/ and this study), we show that the BDU posterior process extends all the way to the mid-body position and then turns towards the ventral-lateral nerve, where PLM runs. The functional properties of the BDU posterior process are unclear. PLMs are a pair of sensory neurons that transduce touch stimuli in the posterior part of the worm body to guide forward movement [28]. Each PLM neuron has its cell body located in the tail region and sends out an anterior process to the mid-body region of the animal. Before reaching the vulva region, this anterior neurite bifurcates. One branch goes ventrally and forms *en passant* chemical synapses with neurons in the ventral nerve cord (Figure 1A “b” region). The other extends continuously forward and terminates anterior of the vulva at a relatively fixed position (Figure 1A “a” region). The neural connection and the function of this anterior lateral branch are not known. When we examined nervous system organization using a cellular green fluorescent protein (GFP) marker (P*unc-86*::MYR::GFP) that highlights both BDU and PLM neurons, we found that the BDU posterior process touches the anterior lateral branch of the PLM neuron. To visualize the BDU and PLM junction region unambiguously, we simultaneously introduced into worms two fluorescent markers, P*unc-53*::GFP and P*mec-7*::mCherry, that label BDU and PLM respectively (Figure 1B and 1C). As shown in Figure 1D, the terminus of the BDU posterior process overlaps with the tip of the PLM neuron.

We next followed the development of BDU and PLM neurites. Using the P*unc-86*::MYR::GFP marker, we found that in 73% (n = 45) of newly hatched L1 larvae, the BDU and PLM neurites were already touching each other. We therefore dissected eggs from gravid adults and followed the development of BDU and PLM in living embryos. BDU and PLM neurons are born at about the same time during the embryonic 1.5 to 2 fold stage, around 450 min after the first cell division (Figure 1E). Two hours after birth, the BDU neuron has completed its posterior migration and leaves an anterior neurite behind (∼570 min after the first cell division). No posterior BDU process, however, can be seen by the end of this two-hour period. In contrast, the PLM cell starts sending out the anterior neurite once it is born, and by ∼570 min this neurite has already grown to a significant length (Figure 1F). After its posterior migration is finished, the BDU neuron begins to project the posterior neurite (Figure 1G). By the end of embryonic elongation or during the early L1 stage, the posterior BDU neurite is in touch with the PLM anterior process (Figure 1H).

We then tested whether PLM influences BDU neurite outgrowth and *vice versa*. *ced-3* encodes a caspase that is crucial for apoptosis and causes cell death when its expression level is up-regulated [29], [30]. When *ced-3* was over-expressed in PLM using the P*mec-7* promoter, PLM neurons were specifically eradicated, but BDU still projected a single long posterior neurite to the target region (Figure 1I and 1J, n = 29–38). Similarly, in the absence of BDU, PLM still extended a single anterior process just like in wild-type animals (Figure 1K and 1L, n = 25–39). For both BDU and PLM, we found no obvious alteration in cell body position, neurite branching, or the orientation of neurite outgrowth when the target neuron was removed (Figure 1I and 1K). These results indicate that BDU and PLM do not play a significant role in directing long-range growth of the target cell to its destination. Interestingly, we found that when PLM was removed, the BDU process was longer at various developmental stages compared to untreated animals (Figure 1J), suggesting that BDU may have a more active role than PLM in establishing the BDU-PLM connection after the neurites reach the target region.

### Gap junctions form between the BDU and PLM neurons

**Figure 2:**
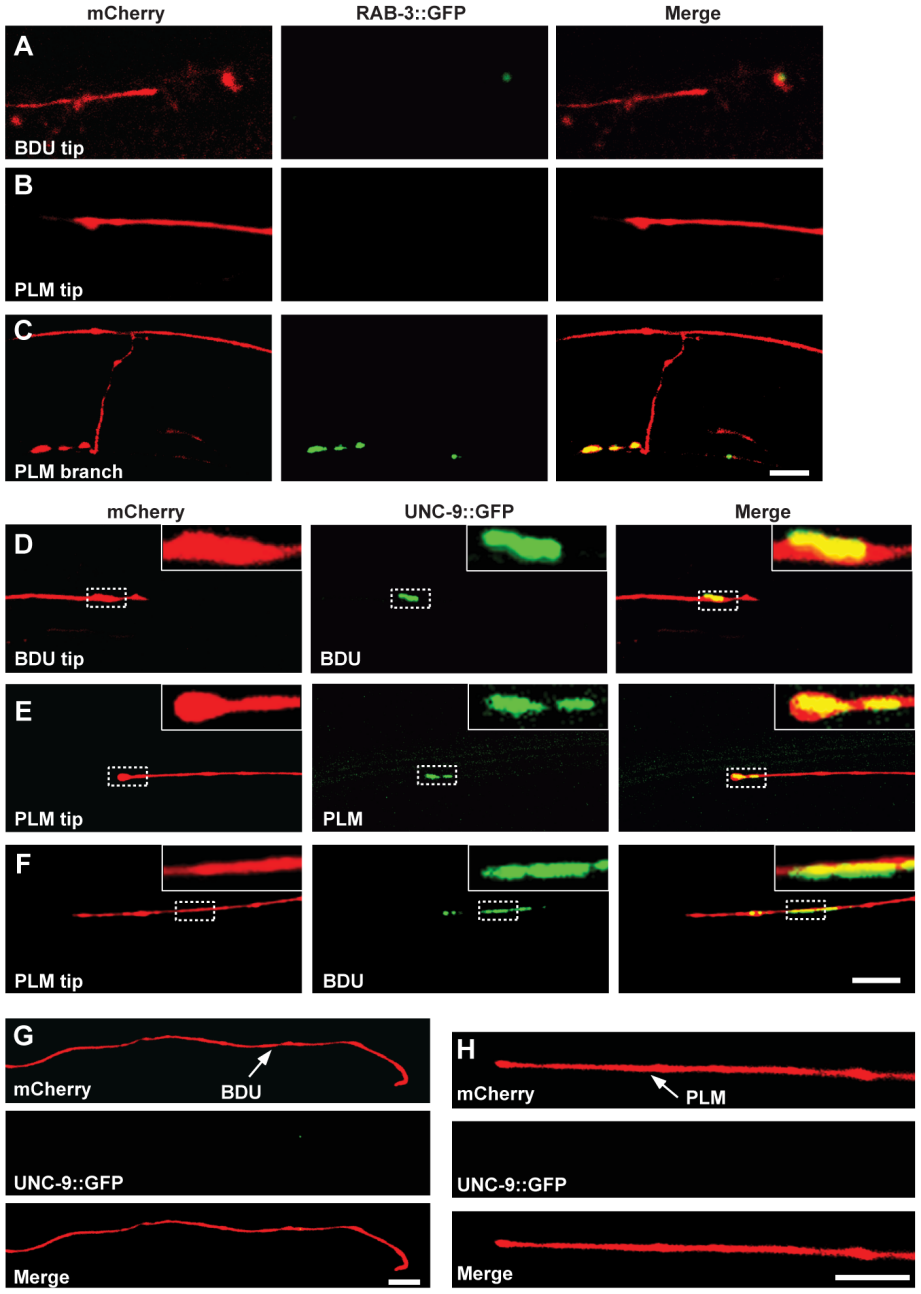
UNC-9::GFP clusters at the interface of BDU and PLM neurons. (A–C) BDU and PLM do not connect through chemical synapses. (A) P*unc-53*::RAB-3::GFP is not present on the BDU posterior tip, which is labeled with P*unc-53*::mCherry. (B) P*mec-7*::RAB-3::GFP is not present on the PLM anterior tip, which is labeled with P*mec-7*::mCherry. (C) RAB-3 (P*mec-7*::RAB-3::GFP) puncta are present on the PLM branch region (b in Figure 1A), which is labeled with P*mec-7*::mCherry. (D–F) BDU and PLM neurons connect through gap junctions. (D) P*unc-53*::UNC-9::GFP, expressed in BDU, colocalizes with the tip of the BDU posterior process, which is labeled with *Punc-53*::mCherry. (E) P*mec-7*::UNC-9::GFP, expressed in PLM, colocalizes with the tip of the PLM anterior process, which is labeled with *Pmec-7*::mCherry. (F) P*unc-53*::UNC-9::GFP, expressed in BDU, colocalizes with the tip of the PLM anterior process, which is labeled with *Pmec-7*::mCherry. (G) When PLM is removed, the UNC-9::GFP cluster in BDU is greatly diminished. The BDU posterior process is labeled by P*unc-86*::mCherry and UNC-9 is labeled by P*unc-53*::UNC-9::GFP. (H) When BDU is removed, the UNC-9::GFP cluster in PLM is greatly diminished. The PLM posterior process is labeled by P*unc-86*::mCherry and UNC-9 is labeled by P*mec-7*::UNC-9::GFP. Scale bars represent 10 µm.

A nerve cell is functionally connected to its target cell through chemical synapses and/or electrical synapses. To determine the biochemical nature of the BDU-PLM connection, we first tested whether BDU connects with PLM through chemical synapses. One characteristic feature of chemical synapses is the enrichment of synaptic vesicles at the pre-synaptic site. RAB-3 is a synaptic vesicle-associated protein that is widely present in all kinds of neurons [31]. While RAB-3 signal was clearly evident along the ventral nerve cord where PLM forms chemical synapses (Figure 1A “b” region and Figure 2C), no accumulation of RAB-3 puncta was found at the posterior termini of BDU neurons or at the anterior tips of PLM neurons (Figure 2A, 2B and 2C). Thus, BDU and PLM neurons may not connect to each other through chemical synapses.

We then asked whether BDU connects to PLM by forming gap junctions. Gap junctions are complex multi-unit plasma membrane structures that are composed of innexins in worms. The *C. elegans* genome contains 25 innexin genes, among which *unc-9* is one of the few that are widely expressed in the nervous system [32]. We generated a functional UNC-9::GFP fusion protein and expressed it in BDU (P*unc-53*::UNC-9::GFP) or PLM (P*mec-7*::UNC-9::GFP). UNC-9::GFP signal was present in a punctate structure at the terminus of the BDU posterior process and at the tip of the PLM anterior neurite (Figure 2D and 2E). We next labeled PLM neurite and BDU gap junctions simultaneously, and found that the gap junction GFP puncta in BDU co-localized with the tip of the anterior PLM process (Figure 2F). Furthermore, we found that the UNC-9::GFP cluster is absent when BDU or PLM is removed, implying that neuron-neuron contact may be required for assembling the gap junction cluster (Figure 2G and 2H).

**Figure 3:**
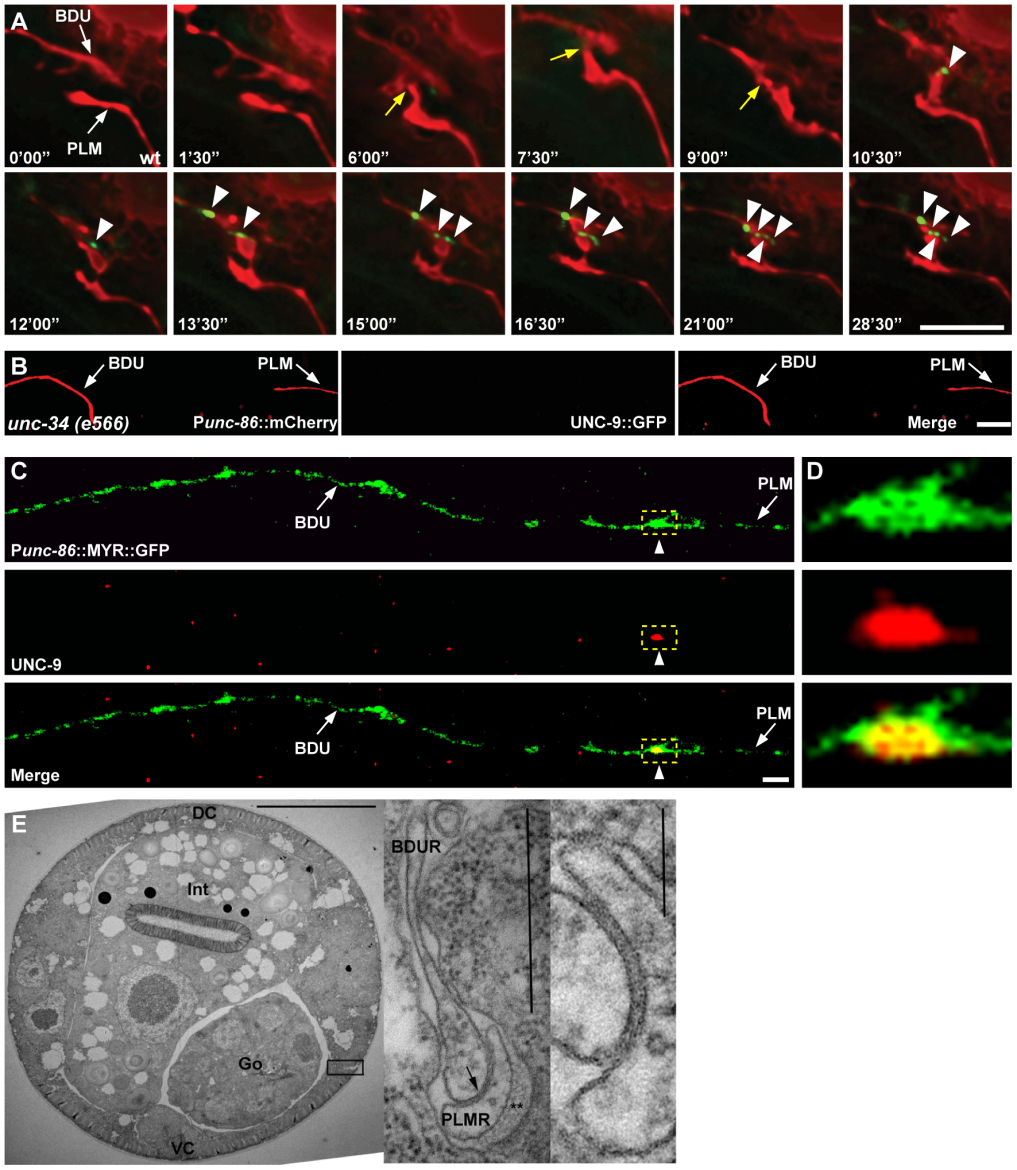
Gap junctions form between BDU and PLM neurons. (A) Time-lapse images of BDU and PLM cells and gap junction plaques. BDU and PLM cells were stained by mCherry. Gap junctions were stained by P*unc-53*::UNC-9::GFP. Images were collected every 90 seconds. Yellow arrows indicate the possible BDU-PLM membrane contact sites. Arrowheads indicate the putative gap junction plaques. (B) No gap junction plaques are formed in unconnected BDU and PLM neurites in *unc-34* mutants. BDU and PLM cells were stained by P*unc-86*::mCherry. Gap junctions were stained by P*unc-86*::UNC-9::GFP. Arrows indicate the BDU and PLM neurites. (C) UNC-9 antibody staining (red) on BDU and PLM cells (labeled green by P*unc-86*::MYR::GFP). Dashed boxes indicate co-localization of UNC-9 with the BDU-PLM junction. (D) Enlarged images of dashed boxes in (C). Scale bars represent 10 µm. (E) Low power TEM image of a wild type L4 animal in the anterior midbody region. Scale bar is 10 µm. Int: intestine; Go: gonad; VC: ventral cord; DC: dorsal cord. Boxed region is shown in the inset on the right, from a nearby section where the posterior BDUR process reaches laterally towards the cuticle to make direct contact with the anterior limit of the PLMR dendrite. Arrow points to a large gap junction. This single junction continued for 37 serial thin sections, roughly 2.5 microns along the body axis. Double asterisk (**) marks the mantle protein that is secreted on the right edge of PLMR, facing the cuticle. One large (15 protofilament) microtubule can be seen in cross-section within the PLMR dendrite; such large diameter microtubules and mantle protein are characteristic only of the “touch dendrites” of ALM, PLM, AVM and PVM. Scale bar in the inset is 0.5 µm. A second inset to the extreme right shows the gap junction at even higher power from another section. Scale bar is 0.1 µm.

Next, we examined during which developmental stage the BDU-PLM gap junction connection is formed. Live imaging showed that the UNC-9::GFP cluster was not detected before the BDU and PLM neurites contacted each other (Figure 3A). Soon after or at the same time that BDU and PLM made contact, the UNC-9::GFP cluster appeared in the neuron-neuron contact region and persisted into adulthood (Figure 3A). In Enabled/VASP mutant *unc-34* animals, which are defective in neurite outgrowth, BDU and PLM failed to contact each other and no UNC-9::GFP cluster could be identified (Figure 3B). The fact that direct membrane contact is required for UNC-9::GFP clustering indicates that BDU and PLM connect through gap junctions. In addition, we used an UNC-9 antibody to detect endogenous gap junctions [33] and found that it highlights punctate structures at the BDU-PLM junction, but not in other regions along the BDU or PLM processes (Figure 3C and 3D).

Finally, we performed electron microscopy (EM) analysis. The anterior process of PLM could be readily identified in any thin section due to the presence of large diameter microtubules within the process, and the secretion of an electron dense “mantle” protein into the space between this process and the surrounding hypodermis. Except for the gap junction found at the PLM anterior tip (as shown in Figure 3E), this portion of the PLM neurite showed no other synaptic interactions, and never encountered another neuron process except for a few passing circumferential commissures that run periodically between the ventral and dorsal nerve cords, and which generally pass along a different route, virtually orthogonal to the orientation of BDU's posterior process in Figure 1A. Together with the UNC-9 antibody staining pattern and UNC-9::GFP localization, this EM data further support the notion that BDU and PLM neurons indeed form gap junctions.

### Wnt signaling is involved in BDU-PLM target identification

Next, we took both forward and reverse genetic approaches to dissect the genetic regulation of BDU-PLM contact formation. It has been previously reported that in *C. elegans* the gap junction components UNC-7 and UNC-9 are required in the AVB interneuron and the B class motor neuron, respectively, for gap junction clusters to form between these neurons [34]. However, it is unclear whether the expression of UNC-7 and UNC-9 affects the contact between these neurons. We therefore tested whether gap junction components are required for BDU-PLM target identification. Among the 25 *C. elegans* innexins, UNC-7 has been shown to localize in BDU cells [34], while INX-7 and UNC-9 are expressed in both BDU and PLM cells [32]. When we examined *unc-7*, *unc-9*, *inx-7*, *unc-9 unc-7*, and *unc-9*;*inx-7* mutants, we found that BDU and PLM still made contact (Table S1). In addition, UNC-9::GFP clustered at the interface between BDU and PLM in *unc-7* and *inx-7* animals (Figure S1A). Since it is difficult to rule out the possibility that other innexins may be present in BDU and/or PLM cells or that multiple innexins may function redundantly, it remains to be determined whether gap junction components can direct BDU-PLM target identification.

Another possibility is that cell adhesion molecules may facilitate BDU-PLM recognition. We examined a panel of mutations in genes encoding cell adhesion molecules, but none displayed obvious defects in BDU-PLM target identification (Table S1).

**Figure 4:**
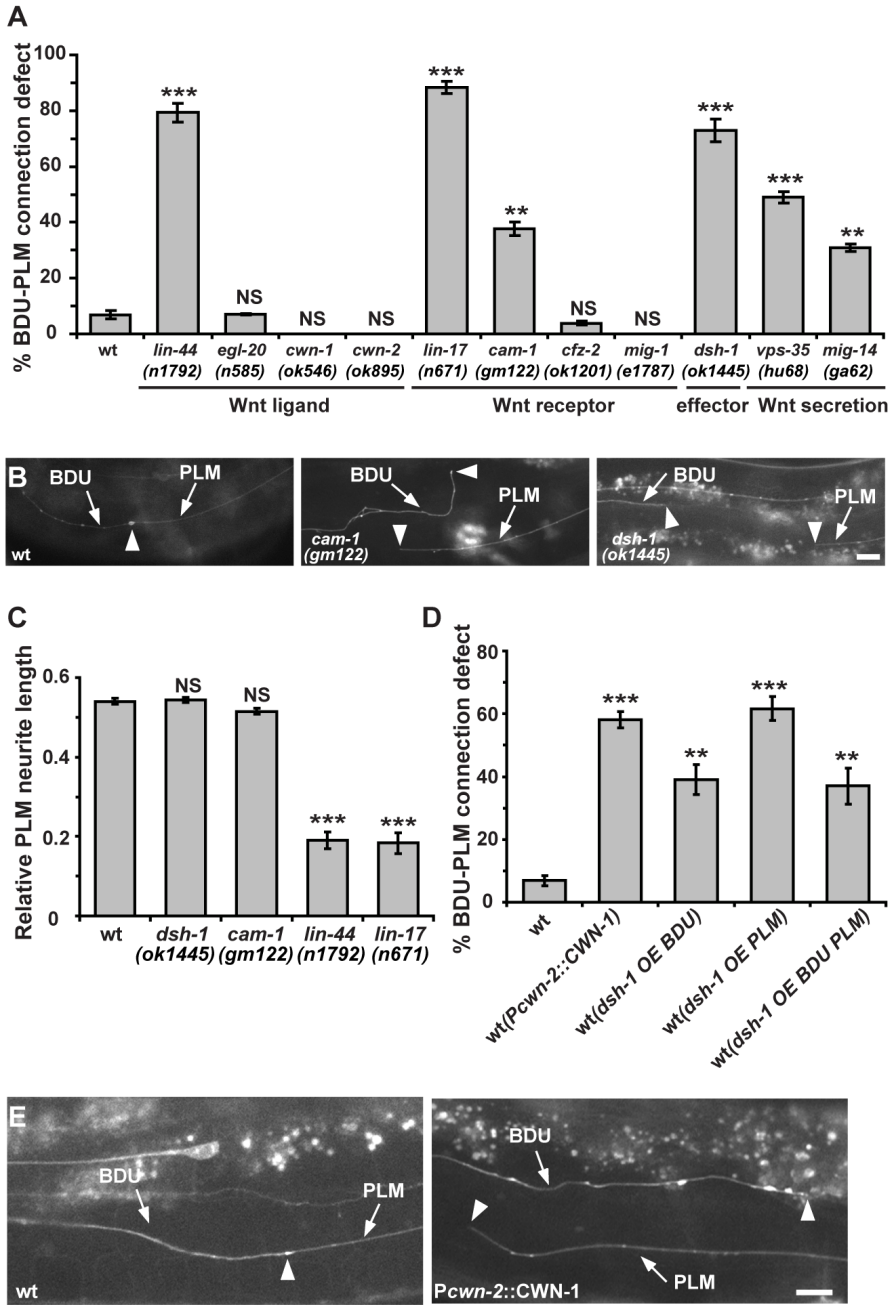
The Wnt pathway is involved in BDU-PLM connection. (A) Quantification of BDU-PLM connection defects in various mutants in the Wnt pathway. (B) The defective BDU-PLM contact phenotype in *cam-1* and *dsh-1* mutants. Arrows indicate the BDU and PLM processes. Arrowheads point to the contact region (left panel) or neurite tips (middle and right panels). (C) While *lin-44* and *lin-17* affect the polarity of PLM, resulting in shorter PLM neurites, the PLM neurite length is not reduced in *dsh-1* and *cam-1* mutants. (D) Ectopic CWN-1 expression (P*cwn-2*::CWN-1) or over-expression of DSH-1 (OE) in BDU (P*unc-53*::DSH-1) or PLM (P*mec-7*::DSH-1) or both BDU and PLM (co-injected with both P*unc-53*::DSH-1 and P*mec-7*::DSH-1) causes BDU-PLM contact defects. Error bars in (A, C and D) represent the SEM. *** p<0.001; ** p<0.01; NS, not significant. (E) In wild type, the BDU posterior process contacts the PLM anterior process. The arrowhead indicates the BDU-PLM junction. When CWN-1 is ectopically expressed using P*cwn-2*, BDU and PLM fail to contact. Arrowheads indicate the tips of the BDU and PLM neurites. Scale bars represent 10 µm.

We then examined a panel of molecules that have been implicated in axon guidance, including components of the Netrin, Slit, Ephrin, and Wnt pathways. In *unc-6*/*Netrin*, *unc-40*/*DCC*, *slt-1*/*Slit*, *sax-3*/*Robo*, and *vab-1*/*Ephrin receptor* mutant animals, the BDU and PLM neurons were still able to contact each other (Figure S1B). In contrast, several mutants in the Wnt pathway, including *lin-44*/*Wnt*, *lin-17*/*Wnt receptor*, *cam-1*/*Wnt co-receptor* and *dsh-1*/*Dishevelled*, displayed defective BDU-PLM contact (Figure 4A, 4B and Figure S1C). In addition, mutations in *mig-14* and *vps-35*, which act upstream to regulate the secretion of Wnts [35], [36], also cause failure of BDU-PLM contact (Figure 4A). Previous studies showed that PLM polarity is influenced by LIN-44/Wnt signaling [36], [37]. Indeed, in the absence of LIN-44/Wnt or its receptor LIN-17/Frizzled, the PLM process is much shorter (Figure 4C). In these mutants, the abnormal BDU-PLM contact may result from cell polarity defects and thus is likely a secondary effect. *dsh-1* appears not to affect the polarity of PLM or the length of the anterior PLM neurite, which is indistinguishable from wild type (Figure 4C). However, the BDU neurite length is greatly reduced in *dsh-1* mutants (Figure S1D), suggesting that the Wnt signal is required for BDU outgrowth.

We also examined the role of CAM-1, the *C. elegans* homolog of the CRD domain-containing receptor tyrosine kinase Ror2 [38]. We previously showed that through its intracellular domain, CAM-1 binds to DSH-1 and serves as a Wnt co-receptor in axon outgrowth [21]. CAM-1 has also been implicated in attenuating Wnt signaling during cell migration and vulval development [18], [19]. In *cam-1* mutants, both BDU and PLM display normal neuronal polarity and neurite length (Figure S1C). However, in 38% (n = 96) of *cam-1(gm12*2) null mutants, BDU failed to contact PLM (Figure 4A and 4B). *cam-1* has been reported to partially affect BDU cell migration [38], but the non-connection phenotype is not entirely correlated with the anterior over-migration defect and 17% of animals (n = 136) with normal BDU cell body position displayed defective BDU-PLM connection. In addition, *cwn-1* and *cwn-2* mutants show BDU migration defects, but the BDU-PLM contacts are normal (Figure 4A) [39]. Together, these results suggested that *cam-1* may affect target identification by BDU and PLM neurons.

Five Wnts, positioned at various locations along the body axis in *C. elegans*, likely cooperate with each other to regulate tissue development [40]. The maternal lethality of *mom-2* precludes loss-of-function analysis. Single mutants of the four other Wnt ligands have either no phenotype or polarity defects (Figure 4A). To address whether Wnt signaling can influence the BDU-PLM contact, we perturbed the distribution of Wnt by over-expression. Previous studies showed that CWN-2 is the only Wnt that is highly expressed in the anterior region of the worm [20], so we used the *cwn-2* promoter to ectopically increase the expression level of the other four Wnts (CWN-1, EGL-20, LIN-44, and MOM-2) individually in the anterior region. We found that ectopic expression of EGL-20, LIN-44, CWN-2, and MOM-2 did not lead to any BDU-PLM connection defect (Figure S1E). In contrast, when CWN-1, a Wnt normally expressed in the middle and posterior of the worm body, was expressed in the anterior part using the *cwn-2* promoter, both BDU and PLM extended neurites long enough to reach each other, but these neurites failed to contact (Figure 4D and 4E). In addition, over-expression of the downstream effector DSH-1 in both BDU and PLM caused BDU-PLM contact failure, while cell polarity and neurite outgrowth of BDU and PLM were normal (Figure 4D and Figure S1F). We further restricted *dsh-1* over-expression to either BDU or PLM and found that this also caused BDU-PLM connection failure (Figure 4D). These observations suggested that the responsiveness of both BDU and PLM to extracellular Wnt signaling must be tightly regulated to ensure precise neuronal connectivity.

### The BDU-PLM contact requires *aha-1* and *ahr-1*

**Figure 5:**
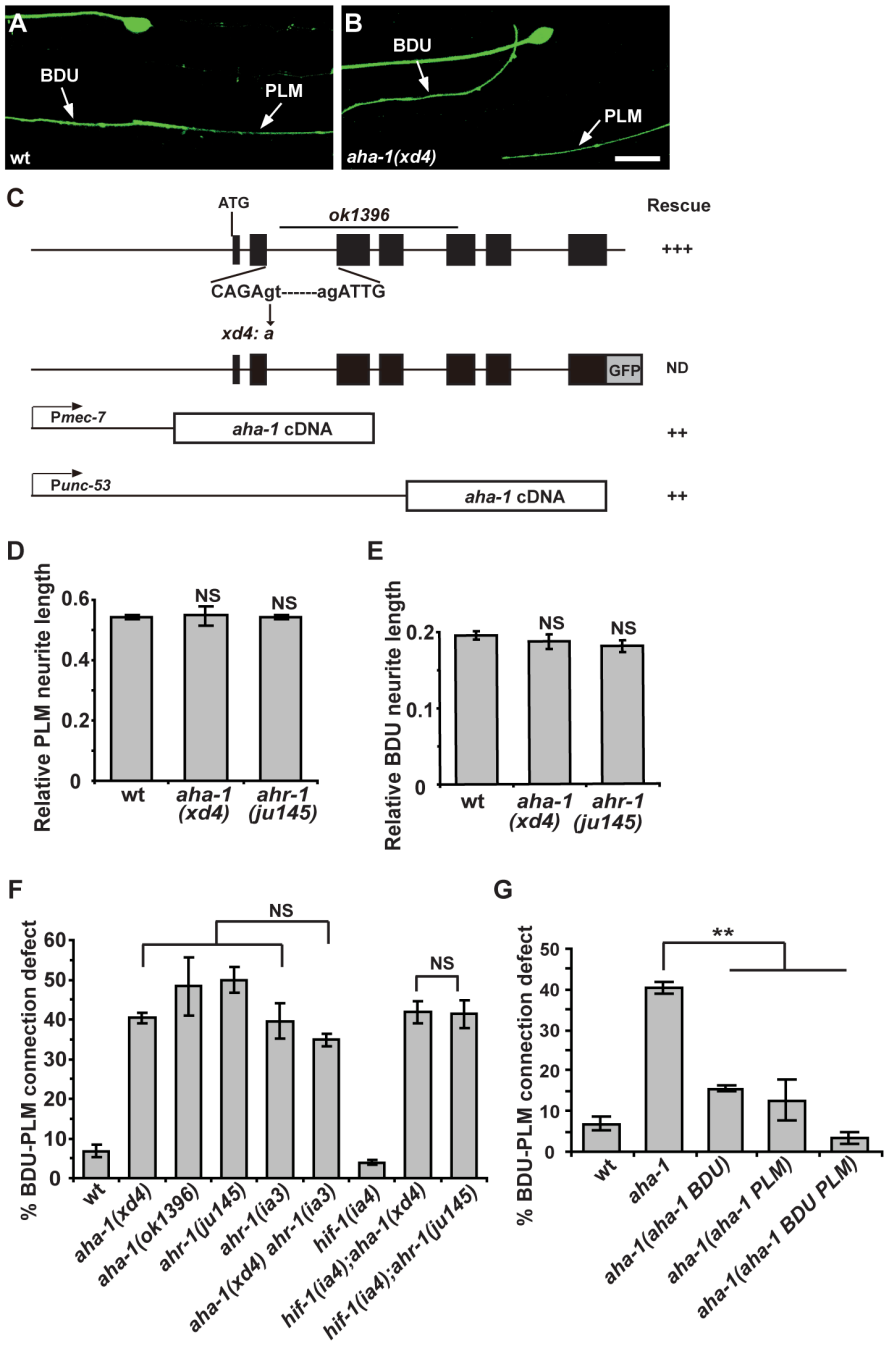
*aha-1* and *ahr-1* are required cell-autonomously for BDU-PLM contact. (A–B) The contact between BDU and PLM is defective in *aha-1(xd4)* animals compared to wild type. Scale bars represent 10 µm. (C) The gene structure of *aha-1*. The molecular lesions in *xd4* and *ok1396* are labeled. The rescue activity of various *aha-1* constructs is indicated. (D–E) PLM and BDU neurite lengths are indistinguishable from wild type in *aha-1(xd4)* or *ahr-1(ju145)* mutants. (F) Quantification of BDU-PLM contact defects in mutants of the PAS-bHLH family. The severity of BDU-PLM contact defects is not enhanced in *aha-1(xd4) ahr-1(ia3)* double mutants compared to *aha-1(xd4)* or *ahr-1(ia3)* single mutants. (G) The *aha-1* mutant phenotype is partially rescued by expressing the *aha-1* gene in BDU (P*unc-53*::AHA-1) or PLM (P*mec-7*::AHA-1) cells and fully rescued by expressing it in both BDU and PLM (P*unc-53*::AHA-1 and P*mec-7*::AHA-1) cells. Error bars in (D–G) represent the SEM. NS, not significant. ** p<0.01.

To reveal the intrinsic molecular machinery responsible for BDU-PLM connection, we performed a genetic screen for mutants which display BDU-PLM contact defects. From the screen, we isolated a mutant named *xd4*. *xd4* animals generally show wild-type morphology and locomotion, but the BDU-PLM contact is lost (Figure 5A and 5B). Through genetic mapping and transgenic rescue experiments, we found that *xd4* is an allele of the *aha-1* gene. We identified a G to A nucleotide transition at the second splice donor site in *xd4* genomic DNA that results in decreased transcription of the *aha-1* gene (Figure 5C and Figure S2A). In *xd4* mutants the lengths of BDU and PLM neurites are indistinguishable from wild-type animals (Figure 5D and 5E), suggesting that the *xd4* mutation may specifically affect the target identification process. The *aha-1(ok1396)* allele contains a large deletion (Figure 5C) and causes an early larval arrest phenotype. *ok1396* larvae show a similar percentage of defective BDU-PLM contacts to *xd4* mutants (Figure 5F). Thus, *xd4* may represent a strong loss-of-function allele of *aha-1*.

*aha-1* encodes the sole *C. elegans* homolog of ARNT (AHR nuclear translocator), which contains a bHLH (basic helix-loop-helix) domain at the N-terminus and a PAS (Per/ARNT/Sim) domain in the middle. ARNT usually forms heterodimers with other PAS domain proteins to regulate gene expression [41]. It has been previously reported that AHA-1 and its partners function together to control neuronal development including neural fate determination [42], [43]. There are five PAS-bHLH family members (AHA-1, AHR-1, HIF-1, CKY-1, and HLH-34) in *C. elegans* [44]. To address which PAS-bHLH protein is the partner of AHA-1 in regulating BDU-PLM contact, we first evaluated the expression profiles of the corresponding genes. Using a promoter-driven GFP assay, we showed that *cky-1* and *hlh-34* were restricted to specific tissues or cells, but not BDU or PLM (Figure S2B and S2C). *hif-1* has been shown to be expressed in every somatic cell [44], while *ahr-1* has been reported to be present in both BDU and PLM neurons [43]. We then examined the loss-of-function phenotypes of *ahr-1*, *hif-1*, *cky-1*, and *hlh-34* mutants or RNAi-treated animals. We found that *ahr-1* mutants display a phenotype similar to *aha-1* mutants, while loss-of-function of *hif-1(ia4)*, *cky-1(RNAi)*, or *hlh-34(RNAi)* did not lead to any detectable phenotype (Figure 5D–F and Table S1). Furthermore, *aha-1 ahr-1* double mutants show a similar phenotype to *aha-1* or *ahr-1* single mutants (Figure 5F), suggesting that *ahr-1* may function together with *aha-1* in formation of the BDU-PLM contact.

Since *aha-1* and *ahr-1* can affect neural cell fate [42], we asked whether the BDU-PLM contact defect in *aha-1* and *ahr-1* mutants is due to cell fate changes. *unc-86* has been shown to be expressed in BDU and PLM, so we used P*unc-86*::MYR::GFP as a BDU-PLM cell-specific marker in this study [45]. We found that in *aha-1* mutants, *unc-86* is still present in BDU and PLM cells (Figure 5A and 5B). The *mec-7* and *mec-18* genes, which are selectively turned on in mechanosensory neurons including PLM, are still expressed in PLM neurons in *aha-1* mutant animals (Figure S2D and S2E). We also used the promoters of two BDU markers, *unc-53* and *nlp-1*, to drive GFP expression in *aha-1* mutants and found that fluorescent signals from both reporters can be detected in BDU (Figure S2F and S2G). Thus, we found no evidence of cell fate changes. Together, these data suggest that *aha-1* and *ahr-1* may ensure that BDU and PLM processes contact one another for the creation of a gap junction connection.

### AHA-1 functions in both BDU and PLM to regulate neuronal connection

Next, we sought to identify the site at which AHA-1 and AHR-1 act during formation of the BDU-PLM connection. A GFP transgene driven by the *aha-1* promoter highlights a wide range of tissues and cells, including neurons, muscle cells, intestine, and epidermis (Figure S2H). We identified the GFP signal in BDU and PLM neurons based on their typical morphology and cell body position (Figure S2I). Consistent with a previous report [44], functional translational AHA-1::GFP is localized in nuclei (Figure S2J). To determine in which tissue or cell *aha-1* function is required, we expressed the *aha-1* cDNA under the control of various cell-specific promoters in *aha-1* mutants. When the *aha-1* transgene was expressed in BDU and PLM together, the BDU-PLM connection defect was fully rescued in almost all animals (Figure 5G), suggesting that *aha-1* functions cell-autonomously to regulate neuronal connection.

We further asked whether *aha-1* acts in BDU or PLM or both. An *aha-1* cDNA driven by the *unc-53* promoter, which confers expression in BDU, partially rescued the *aha-1* mutant phenotype (Figure 5G). Furthermore, when *aha-1* was expressed in PLM, the connection between BDU-PLM was also partially recovered (Figure 5G), suggesting that *aha-1* may function in both BDU and PLM cells to facilitate the formation of BDU-PLM connection.

### AHA-1 directly regulates *cam-1* gene expression

**Figure 6:**
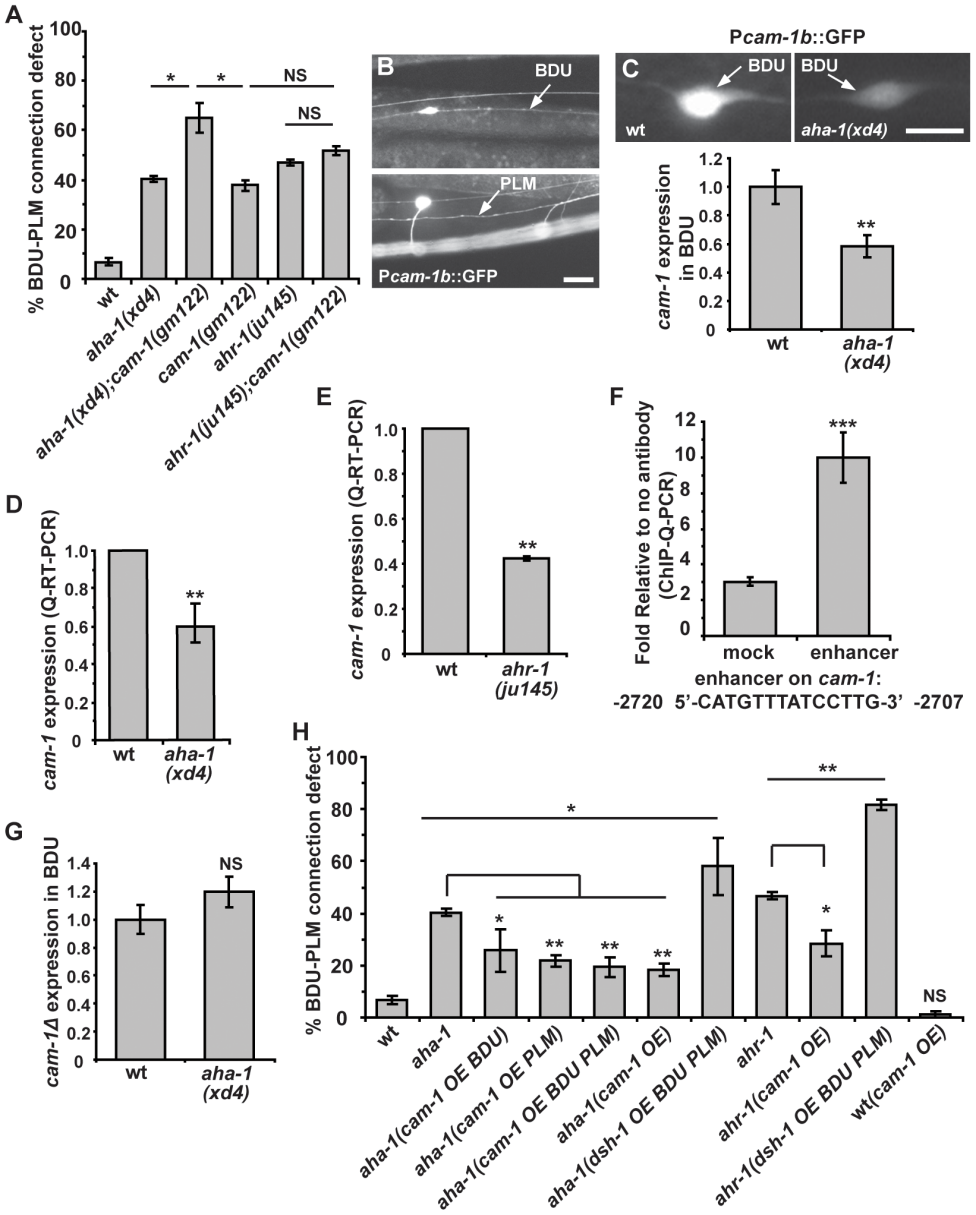
*aha-1* directly regulates *cam-1* gene expression. (A) Quantification of BDU-PLM contact defects in *aha-1(xd4)*, *ahr-1(ju145)*, and *cam-1(gm122)* single and double mutants. (B) P*cam-1b*::GFP is expressed in BDU and PLM cells, as indicated by arrows. (C) Fluorescent images and the GFP fluorescence intensity of P*cam-1b*::GFP in the BDU cell body in wild type and *aha-1(xd4)*. The GFP fluorescence intensity in BDU cell body is measured using ImageJ and the GFP signal in wild type is normalized to 1. Wild type: n = 35; *aha-1(xd4)*: n = 54. (D–E) *cam-1* expression level is decreased in *aha-1* and *ahr-1* mutants. The *cam-1* mRNA level was measured by quantitative RT-PCR and was normalized to 1 in wild type. (F) The association of AHA-1 with the *cam-1* enhancer region is stronger than with the *cam-1* coding region (mock). (G) Expression of GFP driven by P*cam-1bΔ* (*cam-1* promoter lacking the enhancer region) is not affected by mutation of *aha-1*. The GFP fluorescence intensity in BDU cell body is measured using ImageJ and the GFP signal in wild type is normalized to 1. (H) Suppression of BDU-PLM connection defects in *aha-1* and *ahr-1* mutants by over-expression of *cam-1* (OE). Scale bars in (B–C) represent 10 µm. Error bars in (A), (D–F), and (H) represent the SEM. *** p<0.001; ** p<0.01; * p<0.05; NS, not significant.

*cam-1* mutants display a similar phenotype to *aha-1* and *ahr-1* mutants. Therefore, we tested the genetic relationship between *cam-1* and *aha-1* or *ahr-1*. We created double mutants with the *cam-1* null allele *gm112* and the *aha-1(xd4)* allele. In *cam-1;aha-1* double mutants, BDU-PLM contact defects are moderately increased compared to *aha-1(xd4)* or *cam-1(gm112)* single mutants alone (Figure 6A). We further generated *ahr-1; cam-1* double mutants and found that the BDU-PLM contact defect in these animals is similar to that in *ahr-1* null worms (Figure 6A). These results suggest that *cam-1* may act in the same pathway as *aha-1* and *ahr-1*.

Since the AHR/ARNT heterodimer controls gene expression, our results raise the possibility that AHA-1 and AHR-1 may regulate *cam-1* transcription in BDU and PLM. To test this, we compared the *cam-1* expression level in wild type and *aha-1* mutants using GFP reporter and quantitative RT-PCR approaches. In wild-type animals, a GFP reporter driven by a 5.4 kb *cam-1b* promoter sequence is highly expressed in BDU cells (Figure 6B). However, in *aha-1(xd4)* animals, the P*cam-1b*::GFP intensity is decreased (Figure 6C). We also attempted to examine the P*cam-1b*::GFP intensity in PLM, but the cluster of neurons surrounding the PLM region prevented us from carrying out this analysis. We further examined the endogenous *cam-1* mRNA level using quantitative RT-PCR and found that the *cam-1* mRNA level is significantly decreased in *aha-1* mutants compared to wild-type animals (Figure 6D). The *cam-1* mRNA level is also reduced in *ahr-1* mutants (Figure 6E), suggesting that *cam-1* expression is affected by both *aha-1* and *ahr-1*.

Does AHR-1/AHA-1 directly regulate *cam-1* gene expression? The AHR/ARNT heterodimer binds a specific DNA sequence, the Xenobiotic Response Element (XRE, 5′-CACGC-3′), to regulate the transcription of its target genes [41]. However, we searched the 5.4 kb promoter region of *cam-1* and did not identify any obvious XRE sequence. In mammals, AHR/ARNT heterodimers can be recruited specifically to an enhancer element 5′-CATGN6CT/ATG-3′ by unknown factors, thus promoting transcription [46]. Interestingly, a putative AHR/ARNT enhancer sequence 5′-CATGTTTATCCTTG-3′ is found at position -2720 to -2707 relative to the *cam-1* start codon. To test whether AHA-1 indeed regulates *cam-1* expression by binding to this enhancer region, we performed chromatin-immunoprecipitation followed by quantitative PCR (ChIP-Q-PCR) in whole worm extracts. AHA-1::GFP immunoprecipitated by GFP antibody shows significantly higher binding affinity to the putative AHR/ARNT enhancer region than to a coding region sequence in the sixth exon of *cam-1* corresponding to bases 7161-7134 (Figure 6F). We made a GFP construct driven by the *cam-1Δ* promoter in which this enhancer region was deleted. The GFP signal intensity of this reporter was no longer influenced by mutation of *aha-1* (Figure 6G, n = 48 for wild type and n = 53 for *aha-1*), further supporting the notion that AHA-1 may regulate *cam*-1 expression by binding to the enhancer region.

Our genetic analyses indicated that *cam-1* likely negatively regulates Wnt signaling during BDU-PLM connection. Therefore, loss-of-function of *aha-1* and *ahr-1* may create a scenario in which the Wnt signal is enhanced. Consistent with this idea, we found that over-expression of *dsh-1* indeed further enhanced the BDU-PLM connection defects in *aha-1* and *ahr-1* mutants from 40% to 58% and 47% to 82% respectively (Figure 6H). In contrast, increasing the *cam-1* level suppressed the *aha-1* and *ahr-1* mutant phenotypes. As shown in Figure 6H, when *cam-1* was over-expressed using its endogenous promoter, the percentage of animals with BDU-PLM connection defects decreased from 40% (n = 126) to 18% (n = 169) in *aha-1* mutants and from 47% (n = 109) to 28% (n = 119) in *ahr-1* mutants. To determine in which cell the *cam-1* suppression effect is achieved, we restricted *cam-1* expression to both BDU and PLM cells and found that the *aha-1* phenotype was alleviated (Figure 6H). Furthermore, we over-expressed *cam-1* within either BDU or PLM and found that this also suppressed the *aha-1* mutant phenotype, indicating that *cam-1* functions cell-autonomously (Figure 6H). Together, these data strongly suggest that *aha-1* and *ahr-1* regulate the expression of *cam-1* to facilitate the Wnt-mediated fine-tuning of target identification in formation of the BDU-PLM connection.

## Discussion

Here we have shown that the *C. elegans* BDU and PLM neurons are connected by a gap junction, and that a Wnt fine-tuning mechanism is crucial for bringing the mutual target cells into contact during development.

Previous studies using paired recording techniques revealed that in the neocortex, electrical coupling was found exclusively between GABAergic cells of the same class [47], [48]. In *C. elegans*, the posterior process of BDU and the anterior process of PLM contact multiple tissues and cells during development [27], but the gap junction only forms when these two processes meet each other. This high specificity points to a tight regulation of gap junction formation *in vivo*. However, the process by which an individual cell is guided to its appropriate partner with which to form an electrical synapse remains poorly understood.

Since oriented neurite growth precedes any physical contact between BDU and PLM neurons, it is possible that one or both of the participating neurons may provide signals to guide its corresponding target cell. Interestingly, when we eliminated BDU or PLM, the remaining cell was still able to grow relatively normally towards the target area. These phenomena can be interpreted as follows: extracellular environmental cues guide BDU and PLM neurites independently to the correct target area and the participating neurons themselves may then promote neuronal connectivity by locally refining the target identification process.

Intriguingly, although many well-known guidance cues such as Netrin, Slit, and Ephrin are not required for BDU-PLM connectivity, disturbing Wnt resulted in BDU-PLM contact failure. Single loss-of-function mutations of *mom-2*, *cwn-1*, *cwn-2*, or *egl-20* caused either maternal lethality or no defect in BDU-PLM connection, while the *lin-44* mutation severely affected the polarity of PLM, precluding a definite answer to the question of whether Wnt plays an attractive or repulsive role in the BDU-PLM contact process. However, the reduced BDU neurite growth phenotype in *dsh-1* mutants suggests that Wnt signaling may be important to guide BDU neurites to the correct target region. In addition, mis-expression of CWN-1, overexpression of DSH-1, or loss-of-function of *cam-1*, all specifically disrupted the BDU-PLM contact process, indicating that proper Wnt levels are important for the precise targeting between BDU and PLM cells.

How is the Wnt signal integrated within BDU and PLM? We showed that the PAS-bHLH transcription factors AHA-1 and AHR-1 function autonomously within both BDU and PLM to direct neuronal connectivity. Over-expression of *cam-1* bypasses the requirement for AHA-1 or AHR-1. We further revealed that AHA-1 promotes *cam-1* transcription by associating with a *cam-1* enhancer element. These results highlight a local Wnt detection process mediated by the transcription factors AHA-1 and AHR-1. Previous studies have suggested a link between Wnt and AHR. In prostate cancer cells, AHR was identified as a target gene of the Wnt/β-catenin pathway [49]. On the other hand, in liver progenitor cells and MCF-7 cells, AHR activation leads to down-regulation of Wnt/β-catenin signaling [50], [51]. Similarly, in zebrafish, blockage of caudal fin regeneration by the AHR/ARNT pathway activator 2,3,7,8-tetrachlorodibenzo-ρ-dioxin is suppressed by down-regulation of LRP6 [52]. These observations are consistent with our notion that Wnt signaling is regulated by AHR/ARNT.

This fine-tuning of Wnt signal detection through AHA-1/AHR-1-mediated CAM-1 expression regulation may provide a way to direct the process placement, thus to ensure the cell-cell contact between mutual target cells. Transcriptional regulation of Wnt inhibitors has been demonstrated in multiple situations. For instance, the LIM-transcription factor Lhx5 and the homeobox protein Barx1 activate transcription of the secreted Wnt antagonists sFRP1 and sFRP2 in forebrain and gut development respectively [53], [54]. Modifying the expression level of secreted Wnt inhibitors affects many surrounding cells, probably cell-non-autonomously, while regulation of the membrane-bound Wnt antagonist CAM-1 may represent a locally confined, cell type-specific and cell-autonomous mechanism, which is particularly valuable for bringing processes in contact with each other.

What is the biological significance of the BDU-PLM gap junction connection? The anterior neurite of BDU mainly receives chemical synaptic input from the mechanosensory neurons ALM and AVM, which reside in the head region to mediate backward movement in response to touch [27]. The PLM neuron transduces touch stimuli in the posterior part of the worm body to guide forward movement [28]. Four pairs of command interneurons act as the common thread in the neuronal circuit linking mechanosensation to locomotion: AVA and AVD are needed for backward movement, while AVB and PVC are required for forward movement [55]. Here we showed that BDU connects with PLM through gap junctions, while previous studies indicate that BDU also innervates PVC and AVA through chemical synapses [27]. Therefore, BDU likely coordinates touch-responsive backward and forward locomotion through both mechanoreceptor neurons and interneurons (Figure S3).

For more than 50 years, neuronal gap junctions have been known to provide a simple, direct mechanism for information signaling between neurons [56]. Over the past decade, there has been a proliferation of investigations into the structure and function of gap junctions in the nervous system. Single neuron injection with a low molecular weight tracer revealed that gap-junction-mediated coupling is cell type-specific [57]. In the future, we would like to exploit the discovery of the BDU-PLM gap junction to dissect the mechanism of neuron-specific gap junction assembly *in vivo*. With the powerful genetics of *C. elegans*, more comprehensive developmental and functional analyses will shed light not only on the common rules governing how individual neurons identify and reach their mutual targets to form gap junctions, but also on the general principles of nervous system organization.

## Materials and Methods

### *C. elegans* genetics

*C. elegans* strains were maintained on NGM plates under standard conditions as described [58]. Mutants and transgenic fluorescence reporters used in these studies are listed here and Table S2: LGI, *unc-40 (e271)*, *aha-1(xd4)*, *aha-1(ok1396)*, *ahr-1(ju145)*, *ahr-1(ia3)*, *lin-44(n1792)*, *lin-17(n671)*, *mig-1(e1787)*, *xdIs27(*P*unc-53*::UNC-9::GFP, P*odr-1*::GFP); LGII, *cwn-1(ok546)*, *cam-1(gm122)*, *dsh-1(ok1445)*, *mig-14(ga62)*, *vab-1(e2)*, *vps-35(hu68)*; LGIV, *inx-7(ok2319)*, *kyIs262*(P*unc-86*::MYR::GFP, P*odr-1*::dsRed), *egl-20(n585)*, *cwn-2(ok895)*, *wyIs22*(P*unc-86*::RAB-3::GFP, P*odr-1*::dsRed); LGV, *cfz-2(ok1201)*, *hif-1(ia4)*, *jsIs37*[P*mec-7*::SNB-1::GFP, *lin-15(+)*]; LGX, *unc-6(ev400)*, *sax-3(ky123)*, *slt-1(eh15)*, *unc-7(e5)*, *unc-9(fc16)*, *unc-9(e101)*, *uIs25*[P*mec-18*::GFP, *dpy-20(+)*].

The *xd4* mutation of *aha-1* was isolated from *kyIs262* animals treated with EMS. Briefly, the BDU-PLM neurite contact phenotype of F2 progeny was examined under a fluorescence microscope, and mutant animals were recovered to produce progeny. A total of 2,500 mutagenized haploid genomes were screened. *xd4* was mapped to chromosome I. The entire coding region of *aha-1* and all exon-intron boundaries were sequenced by amplifying PCR products of the *aha-1* gene from mutant animals. RNA interference of *cky-1* and *hlh-34* was performed in the RNAi-sensitive strain *rrf-3(pk1426)* mutant as described [59]. For RNA interference, dsRNA was injected into young adult *rrf-3(pk1426);kyIs262* animals at the maximum possible concentration. Progeny laid after the first six hours were scored when they grew to the young adult stage.

### Molecular biology and transgenes

A 4.3 kb *aha-1* genomic DNA fragment containing the promoter and coding region was amplified from N2 genomic DNA to perform the rescue experiment. cDNA clones *of aha-1*, *unc-9*, *dsh-1*, and *cam-1* were obtained from Dr. Yuji Kohara (National Institute of Genetics, Mishima, Japan). For expression in both BDU and PLM cells, the *unc-86* promoter was used. For PLM-specific expression, a 0.85 kb *mec-7* promoter fragment was cloned into the SalI sites of *pPD95.75*. For BDU-specific expression, a 2.8 kb *unc-53* promoter sequence was cloned into the BamHI sites of *pPD95.75*. The cDNAs of *aha-1*, *dsh-1*, and *cam-1* were inserted into *unc-86*, *mec-7* or *unc-53* promoter-containing vectors to create cell-specific expression constructs. A translational AHA-1::GFP fusion was constructed by cloning a PCR fragment containing the entire 4.3 kb promoter and coding region into the GFP expression vector *pPD95.75*. The *aha-1* expression construct was prepared by cloning a PCR fragment containing the entire 1.5 kb promoter into the BamHI site of *pPD95.75*. The *unc-9* cDNA was cloned in-frame into the BamHI site of *pPD95.75* containing the *unc-53* promoter to generate P*unc-53*::UNC-9::GFP. Transgenic animals were generated following standard procedures. In general, plasmid DNAs of interest were used at 1–50 ng/µl, and the co-injection markers P*odr-1*::dsRed, pRF4 or P*odr-1*::GFP at 50 ng/µl. *xdIs27* is an integrated transgenic line of P*unc-53*::UNC-9::GFP and was out-crossed three times before being used for subsequent analysis.

### Immunohistochemistry

Immunohistochemistry was performed with whole-mount worms according to previous report [60]. For immunofluorescence detection of endogenous UNC-9, worms were sequentially stained with anti-UNC-9 and anti-GFP antibody. The UNC-9-specific antibody was kindly provided by Dr. Zhaowen Wang. Mouse monoclonal antibody against GFP (Santa Cruz) was used to detect the P*unc-86*::MYR::GFP. Antibodies against UNC-9 or GFP were used at 1∶100 dilution. FITC conjugated goat anti-mouse and Cy3 conjugated goat anti- rabbit (Earthox) secondary antibodies were used at 1∶100 dilution. Samples were viewed with an IX81 Olympus inverted confocal microscope.

### Electron microscopy

An archival series of serial thin sections of an L4 stage wild-type *C. elegans* was closely viewed over a range of about 50 microns (1000 sections) to follow the anterior extent of the PLM dendrites to their most anterior limits. This series of thin sections comes from the archives of the Sydney Brenner lab at MRC/LMB, Cambridge England., which are now kept for curation in the Hall lab. We infer that the second neuron process in Figure 3E comes from BDU, although no attempt was made to trace it across thin sections. Within those serial sections, PLM dendrites formed no other synapses.

### Image collection and phenotypic quantification

Animals were mounted on 2% agar pads in M9 buffer containing 1% 1-phenoxy-2-propanol and examined by fluorescence microscopy. Fluorescence photographs were taken using a Zeiss Axioimager A1 with AxioCam digital camera and Axiovision rel. 4.6 software (Carl Zeiss) or an IX81 Olympus inverted confocal microscope. Contact was considered defective if the BDU neurite failed to touch the PLM neurite. To measure neurites, eggs were allowed to hatch for 10 min, and newly hatched L1 animals were examined immediately or after incubating on food for different lengths of time. The lengths of neurites and of whole animals were traced from photographs and measured with NIH Image J software. The length of each neurite was traced from the center of the cell body to the tip of neurite. The length of each animal was measured from the anus to the tip of the nose. Images of BDU-PLM contact formation during embryonic stages and time-lapse live images were collected on a Delta-vision Core imaging system (Applied Precision) with an UPLSApo 100×/1.40 NA oil-immersion objective and a Photometrics CoolSnap HQ camera. Deconvolution and analysis of images were performed with Softworx (Applied Precision). The quantification of P*cam-1*::GFP and P*Δcam-1*::GFP intensity was done double-blind with the same extra-chromosomal lines. All the statistical tests were done with two-tailed Student's *t*-tests. Unless otherwise indicated, the n-value in this study is around 50.

### Quantitative RT-PCR

Quantitative RT-PCR was performed using a Stratagene Mx3000P QPCR System. For each reaction, three independent experiments were carried out in triplicate. 2×TransStart Green qPCR Supermix was used according to the manufacturer's instructions. PCR consisted of 40 cycles of 95°C for 30 s, 56°C for 30 s and 72°C for 30 s. A final cycle (95°C, 1 min; 56°C, 30 s; 95°C, 30 s) generated a dissociation curve to confirm a single product. A threshold cycle (ΔCt) value was obtained by subtracting *act-3* Ct values from *cam-1* Ct values. *kyIs262* was used as a reference. ΔΔCt values were derived by subtracting *kyIs262* ΔCt from *aha-1* ΔCt and *ahr-1* ΔCt. The relative expression of *cam-1* was then calculated by 2^−ΔΔCt^. Primers for *cam-1* cDNA amplification were 5′ATCAATAGTGCCGCCAATTC3′ and 5′GTGGAGGTCCGAGATGTTGT3′. Primers for amplification of the internal control *act-3* were 5′TCCAAAGGCTAACCGTGAA3′ and 5′GGAAGCGTAGAGGGAGAGG3′.

### ChIP-qPCR assay

*In vivo* ChIP assays were carried out according to a previous report [61]. Briefly, mixed stages of P*aha-1*::AHA-1::GFP transgenic worms were collected and crosslinked with 2% formaldehyde. After sonication, worm lysates were immunoprecipitated using anti-GFP antibody (Abcam, ab290). The ChIP-DNA and input DNA samples (genomic DNA from the same prep) were subjected to qPCR analysis. A 10 µl PCR reaction with each primer set was run in an Agilent Mx 3000P instrument using TransStart Green qPCR SuperMix (TransGen AQ101) according to the manufacturer's instructions. The PCR program was set as follows: Step 1: 95°C for 10 min; Step 2: 95°C for 30 sec; Step 3: 55°C for 30 sec, Step 4: 72°C for 30 sec. Steps 2–4 were repeated 40 times.

## Supporting Information

Figure S1 BDU-PLM contact in various genetic backgrounds. (A) The UNC-9::GFP cluster is still present at the interface of BDU and PLM in *unc-7* and *inx*-7 mutants. (B) The BDU-PLM contact is not affected in *unc-6*, *unc-40*, *sax-3*, *slt-1* or *vab-1* mutant animals. (C) The cell polarity and neuronal morphology of BDU and PLM are not altered in *cam-1* mutants. (D) The polarity and neuronal morphology of BDU and PLM cells are not altered in *dsh-1* mutants. However, the BDU neurite length is noticeably shorter compared to wild type. (E) Over-expression of CWN-2, EGL-20, LIN-44, or MOM-2 with the P*cwn-2* promoter does not affect BDU-PLM contact. (F) Over-expression of *dsh-1* in PLM (P*mec-7* promoter) and BDU (P*unc-53* promoter) leads to defective BDU-PLM contact but does not change the cell polarity and neuronal morphology of BDU and PLM. The arrowheads indicate the junction of the BDU posterior process and the PLM anterior process. BDU and PLM processes are indicated by arrows. Scale bars represent 10 µm.
(TIF)

Figure S2 The PAS-BHLH family in BDU-PLM contact. (A) RT-PCR analysis indicates that the transcription level of *aha-1* is reduced by the *aha-1(xd4)* allele. (B) P*cky-1*::GFP is expressed in pharyngeal cells. (C) P*hlh-34*::GFP labels several neurons in the head. (D–G) Cell fate-specific markers of BDU or PLM are not altered in *aha-1* animals. (D) P*mec-18*::GFP is expressed in PLM neurons in wild type and *aha-1* animals. Arrows point to PLM neurites. (E) P*mec-7*::GFP is expressed in PLM cells (arrows) in wild type and *aha-1* animals. (F) P*unc-53*::GFP is expressed in BDU cells in wild type and *aha-1* animals. (G) P*nlp-1*::GFP is expressed in BDU cells in wild type and *aha-1* animals. (H) Confocal image of P*aha-1*::GFP in a whole animal. Scale bar represents 100 µm. (I) P*aha-1*::GFP is expressed in PLM and BDU cells, as indicated by arrows. (J) P*aha-1*::AHA-1::GFP is localized in nuclei. The arrow indicates the BDU cell body. Except in H, all scale bars represent 10 µm.
(TIF)

Figure S3 BDU may facilitate the coordination between forward and backward movements upon touch. Schematic illustration of the neural circuit that links mechanosensation to locomotion. AVM, ALM, and PLM are mechanoreceptor neurons. BDU, AVB, PVC, AVA, and AVD are interneurons. DB, VB, DA, and VA are motor neurons. To reduce the complexity, neurons with similar functions or connectivity are grouped, such as AVM/ALM, AVB/PVC, and AVA/AVD.
(TIF)

Table S1 The role of gap junction components, PAS-bHLH family members, and cell adhesion molecules in BDU-PLM contact. Quantification of BDU-PLM contact defects in various mutant strains is shown.
(DOC)

Table S2 Transgenes and strains generated in this study.
(DOC)

## References

[1] Dickson BJ (2002) Molecular mechanisms of axon guidance. Science 298: 1959–1964. 12471249 10.1126/science.1072165

[2] Lyuksyutova AI, Lu CC, Milanesio N, King LA, Guo N, et al (2003) Anterior-posterior guidance of commissural axons by Wnt-frizzled signaling. Science 302: 1984–1988. 14671310 10.1126/science.1089610

[3] Charron F, Stein E, Jeong J, McMahon AP, Tessier-Lavigne M (2003) The morphogen sonic hedgehog is an axonal chemoattractant that collaborates with netrin-1 in midline axon guidance. Cell 113: 11–23. 12679031 10.1016/s0092-8674(03)00199-5

[4] Fannon AM, Colman DR (1996) A model for central synaptic junctional complex formation based on the differential adhesive specificities of the cadherins. Neuron 17: 423–434. 8816706 10.1016/s0896-6273(00)80175-0

[5] Shapiro L, Love J, Colman DR (2007) Adhesion molecules in the nervous system: structural insights into function and diversity. Annu Rev Neurosci 30: 451–474. 17600523 10.1146/annurev.neuro.29.051605.113034

[6] White JG, Southgate E, Thomson JN (1992) Mutations in the Caenorhabditis elegans unc-4 gene alter the synaptic input to ventral cord motor neurons. Nature 355: 838–841. 1538764 10.1038/355838a0

[7] Miller DM, Shen MM, Shamu CE, Burglin TR, Ruvkun G, et al (1992) C. elegans unc-4 gene encodes a homeodomain protein that determines the pattern of synaptic input to specific motor neurons. Nature 355: 841–845. 1347150 10.1038/355841a0

[8] Winnier AR, Meir JY, Ross JM, Tavernarakis N, Driscoll M, et al (1999) UNC-4/UNC-37-dependent repression of motor neuron-specific genes controls synaptic choice in Caenorhabditis elegans. Genes Dev 13: 2774–2786. 10557206 10.1101/gad.13.21.2774 PMC317130

[9] Von Stetina SE, Fox RM, Watkins KL, Starich TA, Shaw JE, et al (2007) UNC-4 represses CEH-12/HB9 to specify synaptic inputs to VA motor neurons in C. elegans. Genes Dev 21: 332–346. 17289921 10.1101/gad.1502107 PMC1785118

[10] Schneider J, Skelton RL, Von Stetina SE, Middelkoop TC, van Oudenaarden A, et al (2012) UNC-4 antagonizes Wnt signaling to regulate synaptic choice in the C. elegans motor circuit. Development 139: 2234–2245. 22619391 10.1242/dev.075184 PMC3357913

[11] Nakamura H, O'Leary DD (1989) Inaccuracies in initial growth and arborization of chick retinotectal axons followed by course corrections and axon remodeling to develop topographic order. J Neurosci 9: 3776–3795. 2585055 10.1523/JNEUROSCI.09-11-03776.1989 PMC6569936

[12] Cheng HJ, Nakamoto M, Bergemann AD, Flanagan JG (1995) Complementary gradients in expression and binding of ELF-1 and Mek4 in development of the topographic retinotectal projection map. Cell 82: 371–381. 7634327 10.1016/0092-8674(95)90426-3

[13] Colon-Ramos DA, Margeta MA, Shen K (2007) Glia promote local synaptogenesis through UNC-6 (netrin) signaling in C. elegans. Science 318: 103–106. 17916735 10.1126/science.1143762 PMC2741089

[14] Zou Y (2004) Wnt signaling in axon guidance. Trends Neurosci 27: 528–532. 15331234 10.1016/j.tins.2004.06.015

[15] Klassen MP, Shen K (2007) Wnt signaling positions neuromuscular connectivity by inhibiting synapse formation in C. elegans. Cell 130: 704–716. 17719547 10.1016/j.cell.2007.06.046

[16] Hikasa H, Shibata M, Hiratani I, Taira M (2002) The Xenopus receptor tyrosine kinase Xror2 modulates morphogenetic movements of the axial mesoderm and neuroectoderm via Wnt signaling. Development 129: 5227–5239. 12399314 10.1242/dev.129.22.5227

[17] Mikels AJ, Nusse R (2006) Purified Wnt5a protein activates or inhibits beta-catenin-TCF signaling depending on receptor context. PLoS Biol 4: e115. 16602827 10.1371/journal.pbio.0040115 PMC1420652

[18] Green JL, Inoue T, Sternberg PW (2007) The C. elegans ROR receptor tyrosine kinase, CAM-1, non-autonomously inhibits the Wnt pathway. Development 134: 4053–4062. 17942487 10.1242/dev.005363

[19] Forrester WC, Kim C, Garriga G (2004) The Caenorhabditis elegans Ror RTK CAM-1 inhibits EGL-20/Wnt signaling in cell migration. Genetics 168: 1951–1962. 15371357 10.1534/genetics.104.031781 PMC1448710

[20] Kennerdell JR, Fetter RD, Bargmann CI (2009) Wnt-Ror signaling to SIA and SIB neurons directs anterior axon guidance and nerve ring placement in C. elegans. Development 136: 3801–3810. 19855022 10.1242/dev.038109 PMC2861721

[21] Song S, Zhang B, Sun H, Li X, Xiang Y, et al (2010) A Wnt-Frz/Ror-Dsh pathway regulates neurite outgrowth in Caenorhabditis elegans. PLoS Genet 6: e1001056. 20711352 10.1371/journal.pgen.1001056 PMC2920835

[22] Ho HY, Susman MW, Bikoff JB, Ryu YK, Jonas AM, et al (2012) Wnt5a-Ror-Dishevelled signaling constitutes a core developmental pathway that controls tissue morphogenesis. Proc Natl Acad Sci U S A 109: 4044–4051. 22343533 10.1073/pnas.1200421109 PMC3306699

[23] Cruciat CM, Niehrs C (2013) Secreted and transmembrane Wnt inhibitors and activators. Cold Spring Harb Perspect Med 3: a015081. 10.1101/cshperspect.a015081 PMC3578365 23085770

[24] Fantauzzo KA, Christiano AM (2012) Trps1 activates a network of secreted Wnt inhibitors and transcription factors crucial to vibrissa follicle morphogenesis. Development 139: 203–214. 22115758 10.1242/dev.069971 PMC3231778

[25] Zhong Y, Wang Z, Fu B, Pan F, Yachida S, et al (2011) GATA6 activates Wnt signaling in pancreatic cancer by negatively regulating the Wnt antagonist Dickkopf-1. PLoS One 6: e22129. 21811562 10.1371/journal.pone.0022129 PMC3139620

[26] Shen C, Nettleton D, Jiang M, Kim SK, Powell-Coffman JA (2005) Roles of the HIF-1 hypoxia-inducible factor during hypoxia response in Caenorhabditis elegans. J Biol Chem 280: 20580–20588. 15781453 10.1074/jbc.M501894200

[27] White JG, Southgate E, Thomson JN, Brenner S (1986) The structure of the nervous system of the nematode Caenorhabditis elegans. Philos Trans R Soc Lond B Biol Sci 314: 1–340. 22462104 10.1098/rstb.1986.0056

[28] Chalfie M, Sulston J (1981) Developmental genetics of the mechanosensory neurons of Caenorhabditis elegans. Dev Biol 82: 358–370. 7227647 10.1016/0012-1606(81)90459-0

[29] Yuan JY, Horvitz HR (1990) The Caenorhabditis elegans genes ced-3 and ced-4 act cell autonomously to cause programmed cell death. Dev Biol 138: 33–41. 2307287 10.1016/0012-1606(90)90174-h

[30] Yuan J, Shaham S, Ledoux S, Ellis HM, Horvitz HR (1993) The C. elegans cell death gene ced-3 encodes a protein similar to mammalian interleukin-1 beta-converting enzyme. Cell 75: 641–652. 8242740 10.1016/0092-8674(93)90485-9

[31] Mahoney TR, Liu Q, Itoh T, Luo S, Hadwiger G, et al (2006) Regulation of synaptic transmission by RAB-3 and RAB-27 in Caenorhabditis elegans. Mol Biol Cell 17: 2617–2625. 16571673 10.1091/mbc.E05-12-1170 PMC1474797

[32] Altun ZF, Chen B, Wang ZW, Hall DH (2009) High resolution map of Caenorhabditis elegans gap junction proteins. Dev Dyn 238: 1936–1950. 19621339 10.1002/dvdy.22025 PMC2732576

[33] Chen B, Liu Q, Ge Q, Xie J, Wang ZW (2007) UNC-1 regulates gap junctions important to locomotion in C. elegans. Curr Biol 17: 1334–1339. 17658257 10.1016/j.cub.2007.06.060 PMC1976340

[34] Starich TA, Xu J, Skerrett IM, Nicholson BJ, Shaw JE (2009) Interactions between innexins UNC-7 and UNC-9 mediate electrical synapse specificity in the Caenorhabditis elegans locomotory nervous system. Neural Dev 4: 16. 19432959 10.1186/1749-8104-4-16 PMC2694797

[35] Coudreuse DY, Roel G, Betist MC, Destree O, Korswagen HC (2006) Wnt gradient formation requires retromer function in Wnt-producing cells. Science 312: 921–924. 16645052 10.1126/science.1124856

[36] Prasad BC, Clark SG (2006) Wnt signaling establishes anteroposterior neuronal polarity and requires retromer in C. elegans. Development 133: 1757–1766. 16571624 10.1242/dev.02357

[37] Hilliard MA, Bargmann CI (2006) Wnt signals and frizzled activity orient anterior-posterior axon outgrowth in C. elegans. Dev Cell 10: 379–390. 16516840 10.1016/j.devcel.2006.01.013

[38] Forrester WC, Dell M, Perens E, Garriga G (1999) A C. elegans Ror receptor tyrosine kinase regulates cell motility and asymmetric cell division. Nature 400: 881–885. 10476968 10.1038/23722

[39] Zinovyeva AY, Forrester WC (2005) The C. elegans Frizzled CFZ-2 is required for cell migration and interacts with multiple Wnt signaling pathways. Dev Biol 285: 447–461. 16109397 10.1016/j.ydbio.2005.07.014

[40] Eisenmann DM (2005) Wnt signaling. WormBook 1–17. 18050402 10.1895/wormbook.1.7.1 PMC4781570

[41] Hahn ME (2002) Aryl hydrocarbon receptors: diversity and evolution. Chem Biol Interact 141: 131–160. 12213389 10.1016/s0009-2797(02)00070-4

[42] Huang X, Powell-Coffman JA, Jin Y (2004) The AHR-1 aryl hydrocarbon receptor and its co-factor the AHA-1 aryl hydrocarbon receptor nuclear translocator specify GABAergic neuron cell fate in C. elegans. Development 131: 819–828. 14757639 10.1242/dev.00959

[43] Qin H, Powell-Coffman JA (2004) The Caenorhabditis elegans aryl hydrocarbon receptor, AHR-1, regulates neuronal development. Dev Biol 270: 64–75. 15136141 10.1016/j.ydbio.2004.02.004

[44] Jiang H, Guo R, Powell-Coffman JA (2001) The Caenorhabditis elegans hif-1 gene encodes a bHLH-PAS protein that is required for adaptation to hypoxia. Proc Natl Acad Sci U S A 98: 7916–7921. 11427734 10.1073/pnas.141234698 PMC35443

[45] Finney M, Ruvkun G (1990) The unc-86 gene product couples cell lineage and cell identity in C. elegans. Cell 63: 895–905. 2257628 10.1016/0092-8674(90)90493-x

[46] Sogawa K, Numayama-Tsuruta K, Takahashi T, Matsushita N, Miura C, et al (2004) A novel induction mechanism of the rat CYP1A2 gene mediated by Ah receptor-Arnt heterodimer. Biochem Biophys Res Commun 318: 746–755. 15144902 10.1016/j.bbrc.2004.04.090

[47] Galarreta M, Hestrin S (1999) A network of fast-spiking cells in the neocortex connected by electrical synapses. Nature 402: 72–75. 10573418 10.1038/47029

[48] Gibson JR, Beierlein M, Connors BW (1999) Two networks of electrically coupled inhibitory neurons in neocortex. Nature 402: 75–79. 10573419 10.1038/47035

[49] Chesire DR, Dunn TA, Ewing CM, Luo J, Isaacs WB (2004) Identification of aryl hydrocarbon receptor as a putative Wnt/beta-catenin pathway target gene in prostate cancer cells. Cancer Res 64: 2523–2533. 15059908 10.1158/0008-5472.can-03-3309

[50] Prochazkova J, Kabatkova M, Bryja V, Umannova L, Bernatik O, et al (2011) The interplay of the aryl hydrocarbon receptor and beta-catenin alters both AhR-dependent transcription and Wnt/beta-catenin signaling in liver progenitors. Toxicol Sci 122: 349–360. 21602191 10.1093/toxsci/kfr129

[51] Zhao S, Kanno Y, Nakayama M, Makimura M, Ohara S, et al (2012) Activation of the aryl hydrocarbon receptor represses mammosphere formation in MCF-7 cells. Cancer Lett 317: 192–198. 22123295 10.1016/j.canlet.2011.11.025

[52] Mathew LK, Sengupta SS, Ladu J, Andreasen EA, Tanguay RL (2008) Crosstalk between AHR and Wnt signaling through R-Spondin1 impairs tissue regeneration in zebrafish. FASEB J 22: 3087–3096. 18495758 10.1096/fj.08-109009 PMC2493445

[53] Peng G, Westerfield M (2006) Lhx5 promotes forebrain development and activates transcription of secreted Wnt antagonists. Development 133: 3191–3200. 16854974 10.1242/dev.02485

[54] Kim BM, Buchner G, Miletich I, Sharpe PT, Shivdasani RA (2005) The stomach mesenchymal transcription factor Barx1 specifies gastric epithelial identity through inhibition of transient Wnt signaling. Dev Cell 8: 611–622. 15809042 10.1016/j.devcel.2005.01.015

[55] Chalfie M, Sulston JE, White JG, Southgate E, Thomson JN, et al (1985) The neural circuit for touch sensitivity in Caenorhabditis elegans. J Neurosci 5: 956–964. 3981252 10.1523/JNEUROSCI.05-04-00956.1985 PMC6565008

[56] Furshpan EJ, Potter DD (1957) Mechanism of nerve-impulse transmission at a crayfish synapse. Nature 180: 342–343. 13464833 10.1038/180342a0

[57] Peinado A, Yuste R, Katz LC (1993) Extensive dye coupling between rat neocortical neurons during the period of circuit formation. Neuron 10: 103–114. 8427699 10.1016/0896-6273(93)90246-n

[58] Brenner S (1974) The genetics of Caenorhabditis elegans. Genetics 77: 71–94. 4366476 10.1093/genetics/77.1.71 PMC1213120

[59] Simmer F, Tijsterman M, Parrish S, Koushika SP, Nonet ML, et al (2002) Loss of the putative RNA-directed RNA polymerase RRF-3 makes C. elegans hypersensitive to RNAi. Curr Biol 12: 1317–1319. 12176360 10.1016/s0960-9822(02)01041-2

[60] Nonet ML, Grundahl K, Meyer BJ, Rand JB (1993) Synaptic function is impaired but not eliminated in C. elegans mutants lacking synaptotagmin. Cell 73: 1291–1305. 8391930 10.1016/0092-8674(93)90357-v

[61] Zhong M, Niu W, Lu ZJ, Sarov M, Murray JI, et al (2010) Genome-wide identification of binding sites defines distinct functions for Caenorhabditis elegans PHA-4/FOXA in development and environmental response. PLoS Genet 6: e1000848. 20174564 10.1371/journal.pgen.1000848 PMC2824807

