# Supplementary figures and images for: Neuronal Target Identification Requires AHA-1-Mediated Fine-Tuning of Wnt Signaling in C. elegans
Source: PLoS Genet. 2013 Jun 27;9(6):e1003618. doi: 10.1371/journal.pgen.1003618 (PMC3694823; doi:10.1371/journal.pgen.1003618)

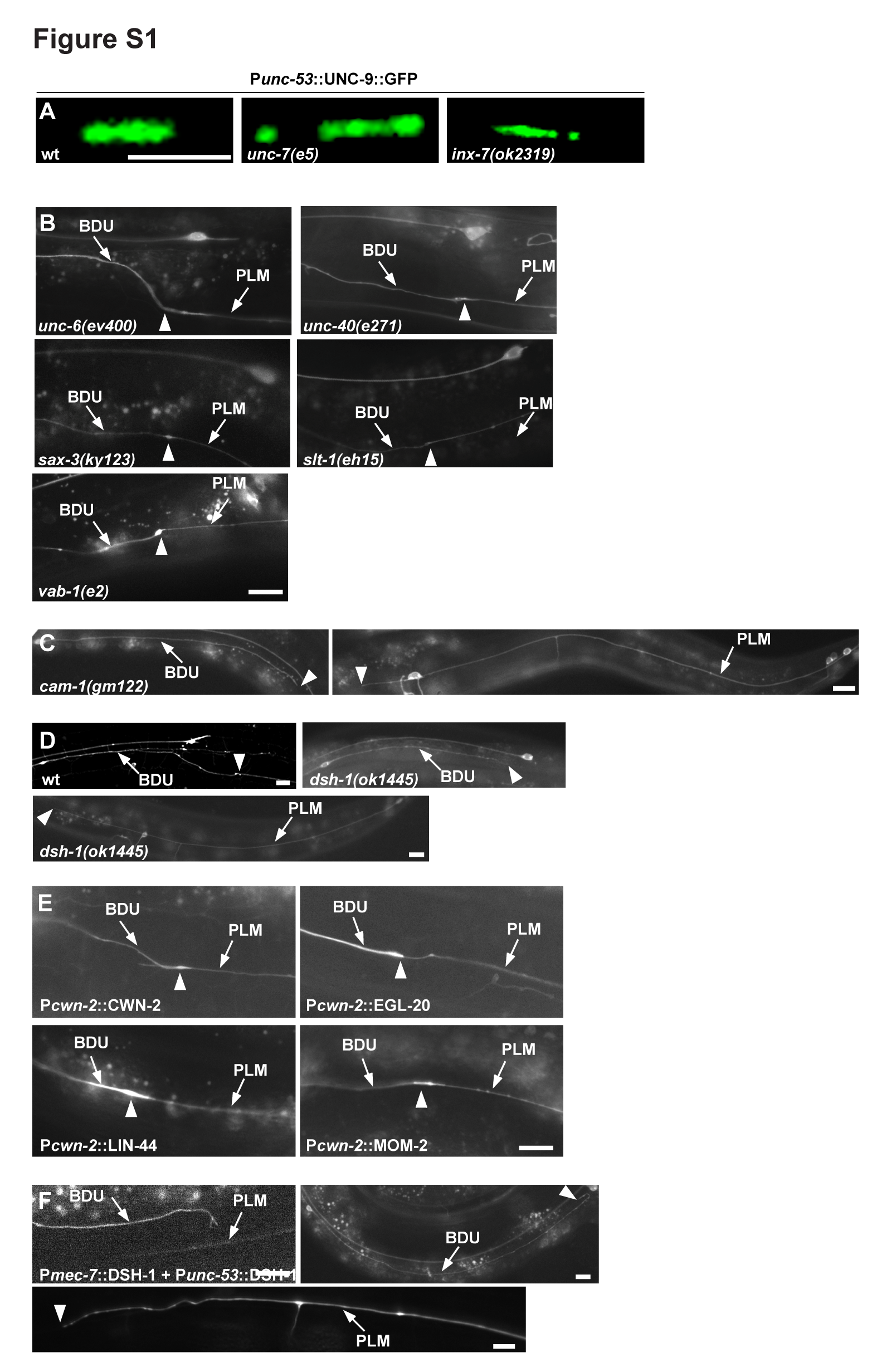

Supplement: Figure S1 — BDU-PLM contact in various genetic backgrounds. (A) The UNC-9::GFP cluster is still present at the interface of BDU and PLM in unc-7 and inx-7 mutants. (B) The BDU-PLM contact is not affected in unc-6, unc-40, sax-3, slt-1 or vab-1 mutant animals. (C) The cell polarity and neuronal morphology of BDU and PLM are not altered in cam-1 mutants. (D) The polarity and neuronal morphology of BDU and PLM cells are not altered in dsh-1 mutants. However, the BDU neurite length is noticeably shorter compared to wild type. (E) Over-expression of CWN-2, EGL-20, LIN-44, or MOM-2 with the Pcwn-2 promoter does not affect BDU-PLM contact. (F) Over-expression of dsh-1 in PLM (Pmec-7 promoter) and BDU (Punc-53 promoter) leads to defective BDU-PLM contact but does not change the cell polarity and neuronal morphology of BDU and PLM. The arrowheads indicate the junction of the BDU posterior process and the PLM anterior process. BDU and PLM processes are indicated by arrows. Scale bars represent 10 µm. (TIF) [file pgen.1003618.s001.tif]

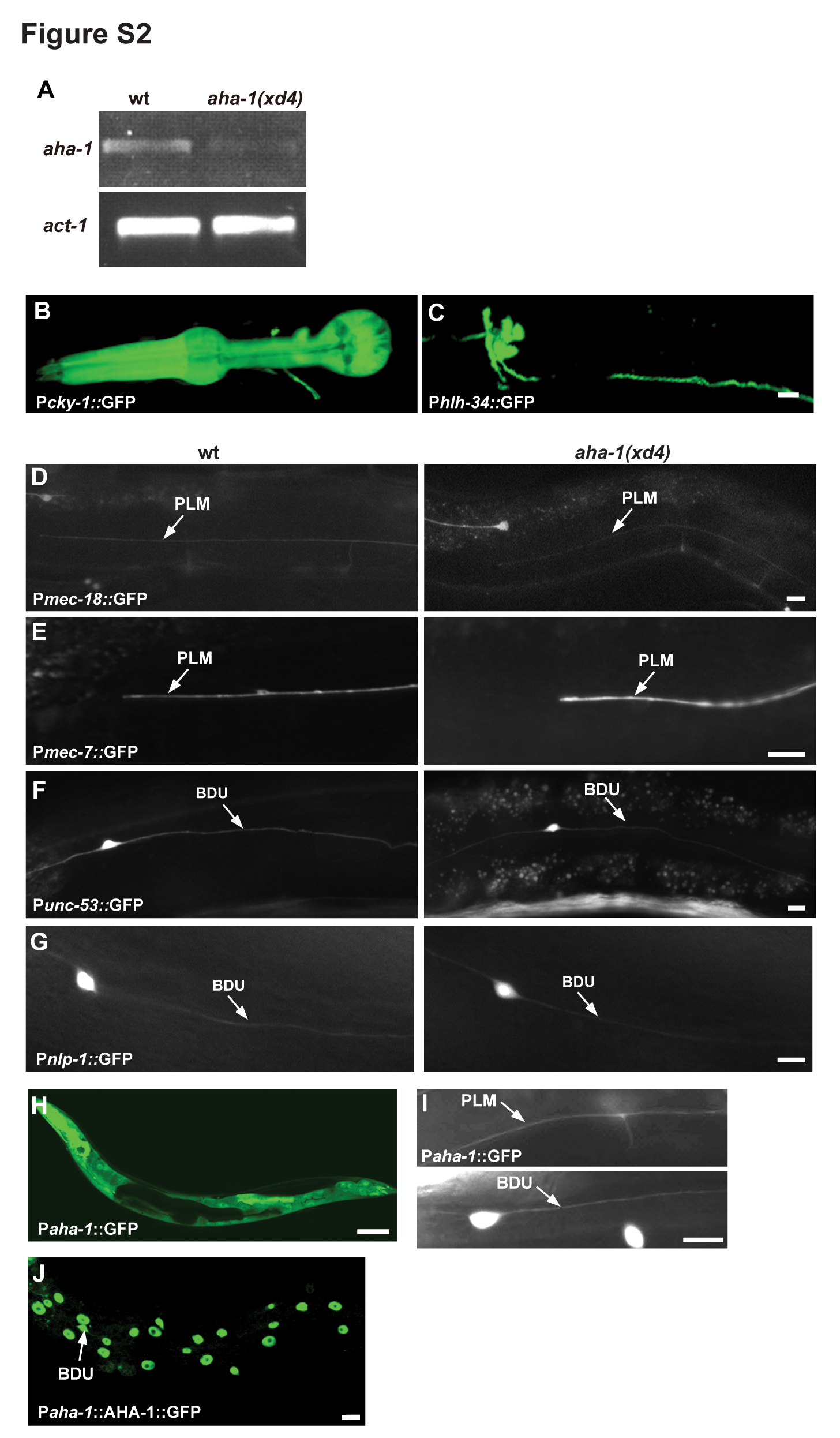

Supplement: Figure S2 — The PAS-BHLH family in BDU-PLM contact. (A) RT-PCR analysis indicates that the transcription level of aha-1 is reduced by the aha-1(xd4) allele. (B) Pcky-1::GFP is expressed in pharyngeal cells. (C) Phlh-34::GFP labels several neurons in the head. (D–G) Cell fate-specific markers of BDU or PLM are not altered in aha-1 animals. (D) Pmec-18::GFP is expressed in PLM neurons in wild type and aha-1 animals. Arrows point to PLM neurites. (E) Pmec-7::GFP is expressed in PLM cells (arrows) in wild type and aha-1 animals. (F) Punc-53::GFP is expressed in BDU cells in wild type and aha-1 animals. (G) Pnlp-1::GFP is expressed in BDU cells in wild type and aha-1 animals. (H) Confocal image of Paha-1::GFP in a whole animal. Scale bar represents 100 µm. (I) Paha-1::GFP is expressed in PLM and BDU cells, as indicated by arrows. (J) Paha-1::AHA-1::GFP is localized in nuclei. The arrow indicates the BDU cell body. Except in H, all scale bars represent 10 µm. (TIF) [file pgen.1003618.s002.tif]

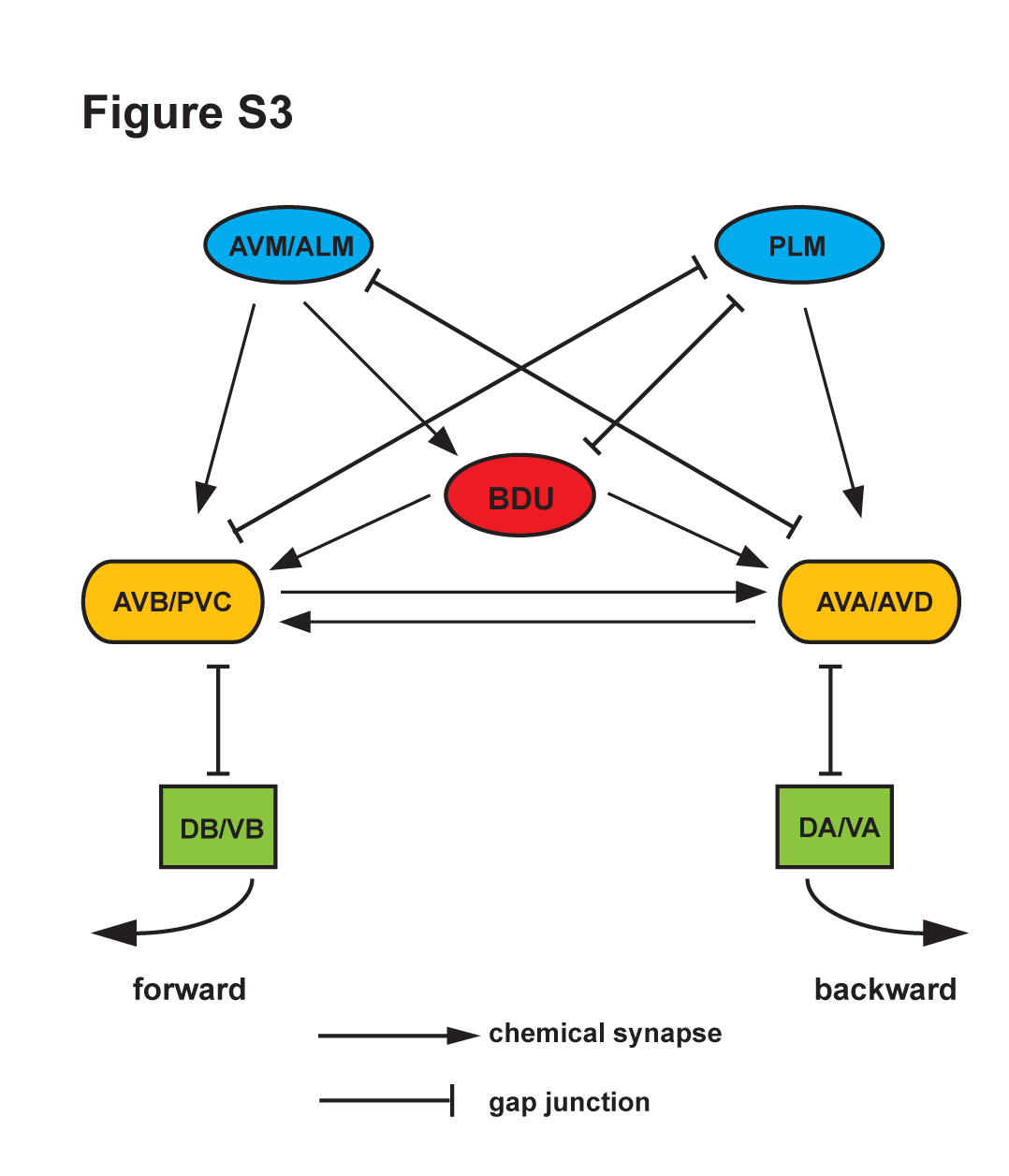

Supplement: Figure S3 — BDU may facilitate the coordination between forward and backward movements upon touch. Schematic illustration of the neural circuit that links mechanosensation to locomotion. AVM, ALM, and PLM are mechanoreceptor neurons. BDU, AVB, PVC, AVA, and AVD are interneurons. DB, VB, DA, and VA are motor neurons. To reduce the complexity, neurons with similar functions or connectivity are grouped, such as AVM/ALM, AVB/PVC, and AVA/AVD. (TIF) [file pgen.1003618.s003.tif]
