# Supplementary material for: Neuronal Target Identification Requires AHA-1-Mediated Fine-Tuning of Wnt Signaling in C. elegans
Source: PLoS Genet. 2013 Jun 27;9(6):e1003618. doi: 10.1371/journal.pgen.1003618 (PMC3694823; doi:10.1371/journal.pgen.1003618)
Supplement: Table S1 — The role of gap junction components, PAS-bHLH family members, and cell adhesion molecules in BDU-PLM contact. Quantification of BDU-PLM contact defects in various mutant strains is shown. (DOC) [file pgen.1003618.s004.doc]

**Table S1.** The role of gap junction components, PAS-bHLH family members, and cell adhesion molecules in BDU-PLM contact

| ***Strain*** | ***Connection defect (%)*** | ***n*** |
| --- | --- | --- |
| Mutants of gap junction components |  |  |
| *unc-7(e5);kyIs262* | 5.3 | 38 |
| *unc-9(e101);kyIs262* | 0 | 20 |
| *inx-7(ok2319);kyIs262* | 4.2 | 24 |
| *unc-9(e101);inx-7(ok2319);kyIs262* | 2 | 50 |
| *unc-9(fc16) unc-7(e5);kyIs262* | 5.8 | 52 |
|  |  |  |
| Mutants of PAS-bHLH family |  |  |
| *cky-1(RNAi);rrf-3(pk1426);kyIs262* | 10.7 | 56 |
| *hlh-34(RNAi);rrf-3(pk1426);kyIs262* | 6.8 | 44 |
|  |  |  |
| Mutants of cell adhesion molecules |  |  |
| *cdh-8(ok628);kyIs262* | 3.8 | 26 |
| *cdh-11(ok739);kyIs262* | 3.1 | 32 |
| *dig-1(n1321);kyIs262* | 3.8 | 52 |
| *egl-15(n484);kyIs262* | 0 | 48 |
| *igcm-1(ok711);kyIs262* | 3.4 | 29 |
| *igcm-2(ok1527);kyIs262* | 2.2 | 45 |
| *ina-1(gm144);kyIs262* | 4.2 | 24 |
| *ketn-1(ok1641);kyIs262* | 2.3 | 43 |
| *mig-6(ev701);kyIs262* | 6.3 | 16 |
| *ptp-3(ok244);kyIs262* | 2.2 | 45 |
| *pxn-1(ok785);kyIs262* | 2.4 | 41 |
| *rig-4(ok1160);kyIs262* | 2.5 | 40 |
| *rig-6(gk376);kyIs262* | 0 | 36 |
| *syg-1(ky652);kyIs262* | 0 | 35 |
| *syg-2(ky673);kyIs262* | 3.6 | 56 |
| *wrk-1(ok695);kyIs262* | 0 | 34 |
| *zig-3(gk33);kyIs262* | 2.5 | 40 |
| *zig-4(gk34);kyIs262* | 2.8 | 36 |
