# Supplementary material for: Neuronal Target Identification Requires AHA-1-Mediated Fine-Tuning of Wnt Signaling in C. elegans
Source: PLoS Genet. 2013 Jun 27;9(6):e1003618. doi: 10.1371/journal.pgen.1003618 (PMC3694823; doi:10.1371/journal.pgen.1003618)
Supplement: Table S2 — Transgenes and strains generated in this study. (DOC) [file pgen.1003618.s005.doc]

**Table S2.** Transgenes and strains

| ***Transgenes*** | ***DNA constructs (concentration)*** | ***Host strains*** |
| --- | --- | --- |
| *xdEx44* | P*cam-1b*::GFP*,* 50 ng/μl | N2 |
| *xdEx124* | P*hlh-34*::GFP*,* 20 ng/μl | N2 |
| *xdEx128* | P*cky-1*::GFP*,* 20 ng/μl | N2 |
| *xdEx306* | P*mec-7*::mCherry*,*15 ng/μl;  P*unc-53*::GFP*,* 50 ng/μl | N2 |
| *xdEx408* | P*mec-7*::AHA-1*,* 20 ng/μl*;*  P*unc-53*::AHA-1*,*50 ng/μl | *aha-1(xd4);kyIs262* |
| *xdEx418* | P*cam-1b*::CAM-1b*,* 10 ng/μl | *aha-1(xd4);kyIs262* |
| *xdEx425* | P*unc-53::*AHA-1*,* 50 ng/μl | *aha-1(xd4);kyIs262* |
| *xdEx428* | P*unc-53*::CAM-1b*,* 1 ng/μl | *aha-1(xd4);kyIs262* |
| *xdEx446* | P*cam-1b*::CAM-1b fosmid*,*10 ng/μl | *ahr-1(ju145);kyIs262* |
| *xdEx452* | P*nlp-1*::GFP*,* 20 ng/μl | N2 |
| *xdEx498* | P*mec-7*::CAM-1b::YFP*,* 20 ng/μl;  *Punc-53*::CAM-1b*,* 1 ng/μl | *aha-1(xd4);kyIs262* |
| *xdEx809* | P*cam-1b*::CAM-1b*,* 2 ng/μl | *kyIs262* |
| *xdEx831* | P*mec-7*::DSH-1*,* 20 ng/μl;  P*unc-53*::DSH-1*,* 50 ng/μl | *aha-1(xd4);kyIs262* |
| *xdEx834* | P*mec-7*::DSH-1*,* 20 ng/μl;  P*unc-53*::DSH-1*,* 50 ng/μl | *ahr-1(ju145);kyIs262* |
| *xdEx836* | P*mec-7*::CAM-1b::YFP*,* 20 ng/μl | *aha-1(xd4);kyIs262* |
| *xdEx844* | P*mec-7::*DSH-1*,10*ng/ul;  P*unc-53*::DSH-1*,10*ng/ul | *kyIs262* |
| *xdEx933* | P*mec-7*::CED-3*,* 20 ng/μl | *kyIs262* |
| *xdEx963* | P*unc-53*::CED-3*,* 50 ng/μl | *kyIs262* |
| *xdEx965* | P*mec-7*::mCherry*,* 20 ng/μl | *xdIs27* |
| *xdEx966* | P*unc-53*::mCherry*,* 60 ng/μl | *xdIs27* |
| *xdEx969* | P*unc-53*::mCherry*,* 50 ng/μl | *wyIs22* |
| *xdEx970* | P*mec-7*::mCherry*,* 20 ng/μl | *wyIs22* |
| *xdEx972* | P*mec-7*::mCherry*,* 20 ng/μl;  P*mec-7*::UNC-9::GFP*,* 20 ng/μl | N2 |
| *xdEx975* | P*mec-7*::AHA-1*,* 20 ng/μl | *aha-1(xd4);kyIs262* |
| *xdEx976* | P*aha-1*::GFP*,* 20 ng/μl | *N2* |
| *xdEx977* | P*mec-7*::DSH-1*,* 20 ng/μl;  P*unc-53*::DSH-1*,* 50 ng/μl | *kyIs262* |
| *xdEx978* | P*unc-53*::DSH-1*,* 50 ng/μl | *aha-1(xd4);kyIs262* |
| *xdEx979* | P*mec-7*::DSH-1*,* 20 ng/μl | *kyIs262* |
| *xdEx993* | P*aha-1*::AHA-1::GFP*,* 20 ng/μl | *N2* |
| *xdEx1119* | P*cwn-2*::CWN-1*,* 30 ng/μl | *kyIs262* |
| *xdEx1248* | P*cam-1b*::GFP∆*,* 50 ng/μl | *N2* |
| *xdEx1250* | P*mec-7*::CED-3*,* 80 ng/μl;  P*unc-86*::mCherry*,* 20 ng/μl | *xdIs27* |
| *xdEx1264* | P*unc-53*::CED-3*,* 80 ng/μl;  P*unc-86*::mCherry*,* 20 ng/μl | *xdEx416* |
| *xdEx1266* | P*mec-7*::mCherry*,* 20 ng/μl | *unc-7* *(e5),xdIs27* |
| *xdEx1267* | P*mec-7*::mCherry*,* 20 ng/μl | *inx-7(ok2319),xdIs27* |
| *xdEx1287* | P*cwn-2*::CWN-2*,* 30 ng/μl | *kyIs262* |
| *xdEx1290* | P*cwn-2*::EGL-20*,* 30 ng/μl | *kyIs262* |
| *xdEx1293* | P*cwn-2*::MOM-2*,* 30 ng/μl | *kyIs262* |
| *xdEx1296* | P*cwn-2*::LIN-44*,* 30 ng/μl | *kyIs262* |
